# Systematic review of learning curves in robot‐assisted surgery

**DOI:** 10.1002/bjs5.50235

**Published:** 2019-11-29

**Authors:** N. A. Soomro, D. A. Hashimoto, A. J. Porteous, C. J. A. Ridley, W. J. Marsh, R. Ditto, S. Roy

**Affiliations:** ^1^ Newcastle Upon Tyne Hospitals NHS Foundation Trust and Newcastle University Newcastle upon Tyne UK; ^2^ Costello Medical London UK; ^3^ Costello Medical Cambridge UK; ^4^ Surgical Artificial Intelligence and Innovation Laboratory, Department of Surgery Massachusetts General Hospital and Harvard Medical School Boston Massachusetts USA; ^5^ Ethicon, Blue Ash Ohio USA

## Abstract

**Background:**

Increased uptake of robotic surgery has led to interest in learning curves for robot‐assisted procedures. Learning curves, however, are often poorly defined. This systematic review was conducted to identify the available evidence investigating surgeon learning curves in robot‐assisted surgery.

**Methods:**

MEDLINE, Embase and the Cochrane Library were searched in February 2018, in accordance with PRISMA guidelines, alongside hand searches of key congresses and existing reviews. Eligible articles were those assessing learning curves associated with robot‐assisted surgery in patients.

**Results:**

Searches identified 2316 records, of which 68 met the eligibility criteria, reporting on 68 unique studies. Of these, 49 assessed learning curves based on patient data across ten surgical specialties. All 49 were observational, largely single‐arm (35 of 49, 71 per cent) and included few surgeons. Learning curves exhibited substantial heterogeneity, varying between procedures, studies and metrics. Standards of reporting were generally poor, with only 17 of 49 (35 per cent) quantifying previous experience. Methods used to assess the learning curve were heterogeneous, often lacking statistical validation and using ambiguous terminology.

**Conclusion:**

Learning curve estimates were subject to considerable uncertainty. Robust evidence was lacking, owing to limitations in study design, frequent reporting gaps and substantial heterogeneity in the methods used to assess learning curves. The opportunity remains for the establishment of optimal quantitative methods for the assessment of learning curves, to inform surgical training programmes and improve patient outcomes.

## Graphical abstract

A broad systematic literature review was performed to characterize the current evidence base and appraise the methods used to measure and define learning curves for surgeons performing robot‐assisted surgery, taking a holistic, panspecialty view. The learning curve estimates identified are subject to considerable uncertainty, and robust evidence was often lacking due to limitations in study design and frequent reporting gaps. Thus, the opportunity remains for the establishment of optimal quantitative methods for the assessment of learning curves, which may inform surgical training programmes and improve patient outcomes.

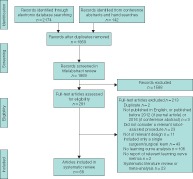

Little consistency between studies

## Abstract (es)

**Antecedentes:**

La aceptación creciente de la cirugía robótica ha generado interés en las curvas de aprendizaje para los procedimientos asistidos por robot. Sin embargo, las curvas de aprendizaje a menudo están mal definidas. Esta revisión sistemática se realizó para identificar la evidencia disponible en relación a las curvas de aprendizaje del cirujano en la cirugía asistida por robot.

**Métodos:**

En Febrero de 2018, se realizaron búsquedas en MEDLINE, Embase y Cochrane Library, de acuerdo con las recomendaciones PRISMA, junto con búsquedas manuales de congresos clave y de revisiones ya existentes. Los artículos elegibles fueron aquellos que evaluaron las curvas de aprendizaje asociadas con la cirugía asistida por robot efectuada en pacientes.

**Resultados:**

Las búsquedas bibliográficas identificaron 2.316 registros de los cuales 68 cumplían los criterios de elegibilidad y correspondían a 68 estudios primarios. De estos 68 estudios, 49 evaluaron las curvas de aprendizaje basadas en datos de pacientes de 10 especialidades quirúrgicas. Los 49 estudios eran todos estudios observacionales, en su mayoría de un solo brazo (35/49 (71%)) e incluían pocos cirujanos. Las curvas de aprendizaje mostraban una notable heterogeneidad, variando entre procedimientos, estudios y parámetros analizados. Los estándares de presentación de informes fueron generalmente deficientes, con solo 17/49 (35%) cuantificando la experiencia previa. Los métodos utilizados para evaluar la curva de aprendizaje fueron heterogéneos, a menudo carecían de validación estadística y usaban terminología ambigua.

**Conclusión:**

Las estimaciones de la curva de aprendizaje estaban sujetas a una considerable incertidumbre, careciendo de evidencia robusta por las limitaciones en el diseño del estudio, lagunas de información en los artículos y heterogeneidad sustancial en los métodos utilizados para evaluar las curvas de aprendizaje. Queda pendiente establecer métodos cuantitativos óptimos para evaluar las curvas de aprendizaje, informar de los programas de formación quirúrgica y mejorar los resultados del paciente.

## Introduction

Learning curves describe the rate of progress in gaining experience or new skills and are widely reported in surgery. Surgeons typically exhibit improvements in performance over time, often followed by a plateau where minimal/limited additional improvement is observed^1^. Generally, surgical learning curves are measured as a change in an operative variable (which can be considered a surrogate for surgeon performance) over a series of procedures. Studies investigating learning curves for surgical procedures are becoming increasingly important, as learning curves can have substantial impact on surgical metrics, clinical outcomes and cost–benefit decisions.

There has been particular interest in learning curves in robot‐assisted surgery, especially in gynaecology and urology^2^, ^3^. Despite the reported operative benefits and improved hospital experience provided by robot‐assisted surgery compared with traditional minimally invasive approaches^4^, ^5^, uptake of robotic technology has been slow, largely due to high capital and maintenance costs, and uncertainty regarding the potential benefits of robot‐assisted approaches over conventional laparoscopic approaches. For example, robot‐assisted approaches have been associated with longer operating times for many procedure types^6^. A large proportion of these comparative studies, however, may have been generated from surgeons who were still learning the robotic technology in question^4^, potentially underestimating the full benefits of robotic assistance. Robot‐assisted approaches have the potential to expedite surgeon learning, but methods used to measure and define learning curves seem inconsistent^1^. Studies evaluating the learning curve for surgical procedures often aim to determine the number of sequential procedures that comprise the learning curve, or that are required to ‘overcome’ the learning curve (sometimes referred to as the learning curve length). To achieve this aim, studies often define a particular threshold in surgeon performance. A common threshold includes reaching a plateau in performance, yet the performance thresholds used are highly inconsistent^1^. The way learning curves are described can lead to misinterpretation. Terms such as ‘overcome’, for instance, could be considered misnomers, implying surgeons have mastered a procedure, for which certain performance thresholds may not provide sufficient evidence. For example, a plateau in performance does not necessarily equate to high‐quality performance; it only implies that a surgeon is no longer improving^1^.

There remains a need to understand better the learning curve of robot‐assisted surgery and broadly characterize how learning curves are defined and reported. This systematic review was performed to characterize the current evidence base and appraise the methods used to define and measure learning curves for surgeons performing robot‐assisted surgery, taking a holistic, panspecialty view.

## Methods

This systematic review was conducted in accordance with a prespecified protocol and the PRISMA guidelines^7^.

MEDLINE, MEDLINE In‐Process, Embase, the Cochrane Database of Systematic Reviews, the Cochrane Central Register of Controlled Trials, and the NHS Economic Evaluation Database were searched. Surgical training has evolved alongside the rapid development of robotic technologies. As such, database searches were limited to the period from 1 January 2012 to 5 February 2018, in order to capture studies investigating learning curves in the context of training relevant to current practice. The search terms used are provided in *Tables* *S1* and *S2* (supporting information). The two most recent abstract books of relevant surgical congresses were also searched from 1 January 2016 to 14 February 2018. This review considered only primary research, and excluded review articles. Supplementary hand searches of the bibliographies of relevant systematic reviews were conducted to identify any primary studies not identified elsewhere.

The review process was performed by two independent reviewers, who assessed the titles and abstracts of all search results (stage 1), as well as the full texts of all potentially eligible studies identified in the first stage (stage 2). In the event of discrepancies, the two reviewers came to a consensus for each decision. In the absence of a consensus, a third independent reviewer resolved any disagreements.

Eligible publications included any randomized or non‐randomized, comparative or observational studies involving wet‐ or dry‐lab testing, simulations, patients or registry/economic analyses that performed a learning curve analysis (consisting of a graph and/or reported data for at least 4 time points) of surgeons performing robot‐assisted surgery in any specialty. Studies were required to report learning curve results from more than one surgeon alone or as part of a surgical team, of any specialization (robot‐assisted, laparoscopic or open). In the absence of reporting the number of surgeons involved, included studies were required to have multiple authors. Only studies that included at least 20 surgical procedures in the analysis of the learning curve were considered. At least one of the following metrics had to be reported: time to plateau/number of ‘phases’ in the learning curve; statistical differences in metrics assessed over time; or learning percentages. Detailed eligibility criteria are shown in *Table* *S3* (supporting information). The studies reported in this review are restricted to learning curve analyses of procedures on patients.

### Data extraction and quality assessment

For each eligible study, data were extracted into a prespecified grid by one reviewer, with verification by a second, independent reviewer. Where there was a discrepancy, the two reviewers attempted to come to a consensus; a third reviewer resolved any disagreements in the absence of a consensus.

Captured data included study design, methodology, surgeon experience, robotic technology used and the metric measured to evaluate the learning curve. Information relating to the learning curve itself was captured, including the number of phases of the curve, the number of operations per phase and the number of procedures to overcome the learning curve (denoted in this review as the point where the chosen performance threshold was considered to have been overcome). Where reported, the specific performance threshold used was captured. If the learning curve had not been overcome within the study period, the number of procedures to overcome the learning curve was reported to be greater than the total number included in the study period.

The quality of each eligible study was assessed using either the UK National Institute for Health and Care Excellence (NICE) RCT checklist^8^ or a modified version of the Downs and Black checklist for non‐randomized studies^9^.

## Results

**Figure 1:**
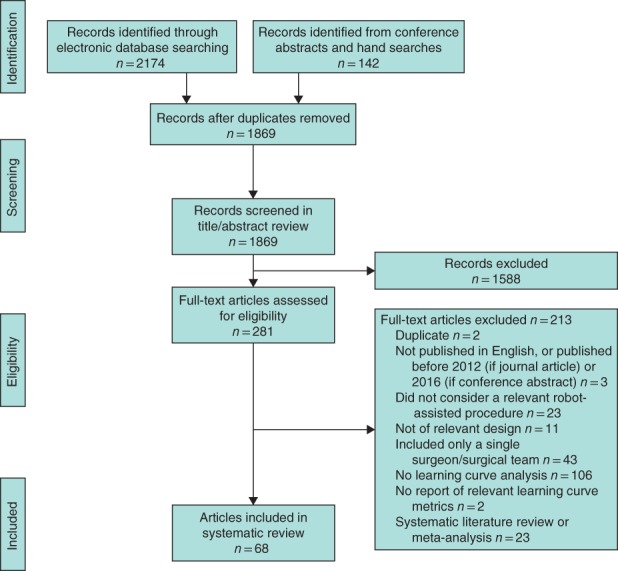
PRISMA diagram for the systematic literature review

A total of 2316 records from electronic database searches, conference abstract searches and hand searches were identified. Of these, 281 full‐text articles were assessed for eligibility, of which 213 were excluded (*Table* *S4*, supporting information). The remaining 68 records (reporting on 68 unique studies) were found to meet the eligibility criteria, 49 of which reported on patient data and are presented here (*Fig*. 1).

### Characteristics of included articles

**Figure 2:**
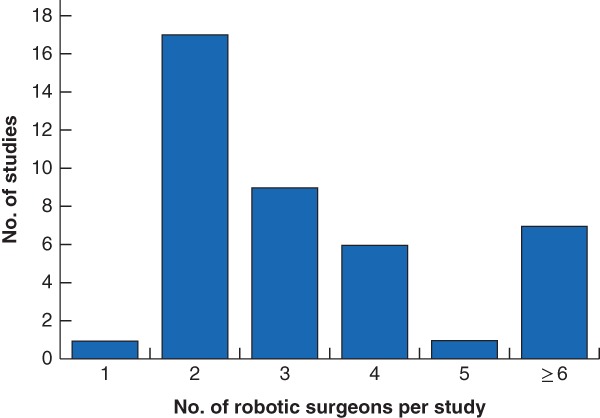
Bar chart showing the number of surgeons in robot‐assisted study arms per study One study enrolled only a single robot‐assisted surgeon, but was considered eligible because the total number of enrolled surgeons was greater than one (the study also evaluated a surgeon who performed procedures laparoscopically).

**Figure 3:**
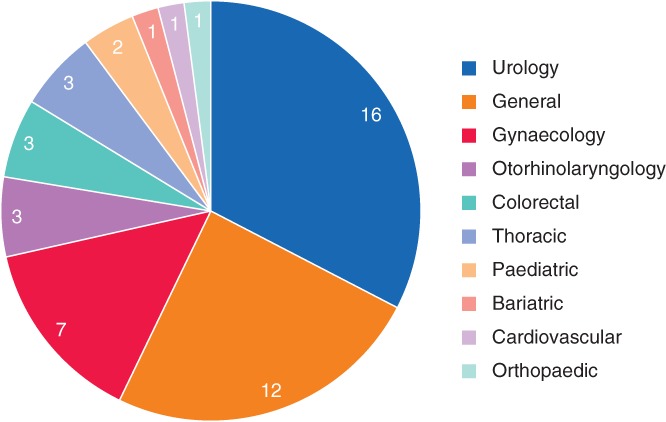
Pie chart of surgical specialties captured in the systematic literature review Values indicate the number of studies involving the specialty.

Characteristics of the 49 eligible studies presenting learning curves derived from patient procedures are presented in *Table* 1 ^10–58^. All were observational in design. Data were analysed retrospectively in 40 of 49 studies (82 per cent), and the remaining nine studies (18 per cent) were prospective in design. The majority (35 of 49, 71 per cent) were single‐arm studies, and the remainder (14 of 49, 29 per cent) were comparative. Among the 41 studies that explicitly defined the number of robotic surgeons per study, the number of participating robotic surgeons was generally small. Most studies (33 of 41, 80 per cent) included fewer than five robotic surgeons, over half of which (18 of 33, 55 per cent) included fewer than three (*Fig*. 2). The captured studies spanned ten surgical specialties (*Fig*. 3). Learning curves were reported most frequently for urology, general surgery and gynaecology.

**Table 1:** Details of studies included in the systematic literature review

Reference	Design	Surgical specialty	Procedure(s)/task(s) performed	Study arms (surgeon experience)*	No. of robotic surgeons
Albergotti *et al*.^10^	Retrospective single‐arm observational	ORL	TORS	Single arm	3
Arora *et al*.^11^	Retrospective single‐arm observational	Urology	RKT	Single arm	n.r.
Benizri *et al*.^12^	Retrospective controlled observational	General	RDP *versus* LDP	RDP	2
Bindal *et al*.^13^	Retrospective single‐arm observational	Bariatric	LRRYGB and TRRYGB	Single arm	2
Binet *et al*.^14^	Retrospective single‐arm observational	Paediatric	Robot‐assisted fundoplications	Single arm	2
Boone *et al*.^15^	Retrospective single‐arm observational	General	RPD	Single arm	4
Chang *et al*.^16^	Retrospective controlled observational	Urology	RARP	RARP (open experience)	1
				RARP (open/laparoscopic experience)	1
				RARP (laparoscopic experience)	1
Ciabatti *et al*.^17^	Prospective single‐arm observational	ORL	Transaxillary robot‐assisted thyroid surgery	Single arm	n.r.
D'Annibale *et al*.^18^	Retrospective single‐arm observational	Urology	Robot‐assisted adrenalectomy	Single arm	n.r.
Davis *et al*.^19^	Retrospective controlled observational	Urology	RARP *versus* ORP	RARP	744
Dhir *et al*.^20^	Retrospective single‐arm observational	General	Robot‐assisted HAI pump placement	Single arm	n.r.
Esposito *et al*.^21^	Retrospective single‐arm observational	Paediatric	REVUR	Single arm	4
Fahim *et al*.^22^	Retrospective single‐arm observational	Thoracic	RATS	Single arm	3
Fossati *et al*.^23^	Retrospective single‐arm observational	Urology	RARP	Single arm	4
Geller *et al*.^24^	Retrospective single‐arm observational	Gynaecology	RSC	Single arm	2
Good *et al*.^25^	Retrospective controlled observational	Urology	RARP *versus* LRP	RARP	1
Goodman *et al*.^26^	Retrospective single‐arm observational	Cardiovascular	Robot‐assisted mitral valve repair	Single arm	2
Guend *et al*.^27^	Retrospective single‐arm observational	Colorectal	Robotic colorectal resection	Single arm	4
Kamel *et al*.^28^	Retrospective single‐arm observational	Thoracic	RAT	Single arm	4
Kim *et al*.^29^	Retrospective controlled observational	Colorectal	RRS	RRS (laparoscopically inexperienced)	1
				RRS (laparoscopically experienced)	1
Lebeau *et al*.^30^	Prospective controlled observational	Urology	RARP	RARP (expert laparoscopic surgeons)	1
				RARP (junior surgeons)	1
Linder *et al*.^31^	Retrospective single‐arm observational	Gynaecology	RSC	Single arm	2
Lopez *et al*.^32^	Retrospective controlled observational	Gynaecology	Robot‐assisted single‐site laparoscopic hysterectomy *versus* LESS hysterectomy	Robot‐assisted single‐site laparoscopic hysterectomy	3
Lovegrove *et al*.^33^	Prospective single‐arm observational	Urology	RARP	Single arm	15
Luciano *et al*.^34^	Retrospective controlled observational	Gynaecology	Robot‐assisted *versus* laparoscopic *versus* vaginal *versus* abdominal hysterectomy	Robot‐assisted hysterectomy	1315
Meyer *et al*.^35^	Retrospective single‐arm observational	Thoracic	Robotic lobectomy	Single arm	2
Myers *et al*.^36^	Retrospective single‐arm observational	Gynaecology	RSC	Single arm	2
Nelson *et al*.^37^	Retrospective single‐arm observational	General	RC	Single arm	8
Odermatt *et al*.^38^	Retrospective controlled observational	Colorectal	Robot‐assisted TME *versus* laparoscopic TME	Robot‐assisted TME	2
Park *et al*.^39^	Prospective single‐arm observational	General	Less than total robot‐assisted thyroidectomy	Single arm	2
Park *et al*.^40^	Retrospective single‐arm observational	General	RAG	Single arm	3
Paulucci *et al*.^41^	Retrospective single‐arm observational	Urology	RAPN	Single arm	2
Pietrabissa *et al*.^42^	Prospective single‐arm observational	General	SSRC	Single arm	5
Pulliam *et al*.^43^	Retrospective controlled observational	Gynaecology	RSC *versus* LSC	RSC	3
Riikonen *et al*.^44^	Retrospective single‐arm observational	Urology	RALP	Single arm	12
Sarkaria *et al*.^45^	Retrospective single‐arm observational	General	RA‐GPEHR	Single arm	n.r.
Schatlo *et al*.^46^	Retrospective single‐arm observational	Orthopaedic	Robot‐assisted placement of pedicle screws	Single arm	13
Shakir *et al*.^47^	Retrospective single‐arm observational	General	RDP	Single arm	3
Sivaraman *et al*.^48^	Retrospective controlled observational	Urology	RARP *versus* LRP	RARP	9
Sood *et al*.^49^	Prospective controlled observational	Urology	RKT	RKT (extensive RKT experience, limited OKT experience)	1
				RKT (extensive RKT and OKT experience)	1
				RKT (limited RKT experience, extensive OKT experience)	1
Tasian *et al*.^50^	Prospective controlled observational	Urology	Robot‐assisted pyeloplasty	Robot‐assisted pyeloplasty (fellow surgeon)	4
				Robot‐assisted pyeloplasty (attending surgeon)	1
Tobis *et al*.^51^	Retrospective single‐arm observational	Urology	RAPN	Single arm	3
van der Poel *et al*.^52^	Retrospective single‐arm observational	Urology	LND during RARP	Single arm	2
Vidovszky *et al*.^53^	Prospective single‐arm observational	General	SSRC	Single arm	n.r.
White *et al*.^54^	Prospective single‐arm observational	ORL	TORS	Single arm	n.r.
Woelk *et al*.^55^	Retrospective single‐arm observational	Gynaecology	Robot‐assisted hysterectomy	Single arm	2
Wolanski *et al*.^56^	Retrospective controlled observational	Urology	RALP *versus* LRP	RALP	2
Zhou *et al*.^57^	Retrospective single‐arm observational	General	RAG	Single arm	2
Zureikat *et al*.^58^	Retrospective single‐arm observational	General	Robot‐assisted pancreatic resections	Single arm	n.r.

* Surgeon experience only stated in studies with multiple robotic study arms. ORL, otorhinolaryngology; TORS, transoral robot‐assisted surgery; RKT, robot‐assisted kidney transplantation; n.r., not reported; RDP, robot‐assisted distal pancreatectomy; LDP, laparoscopic distal pancreatectomy; LRRYGB, laparoscopic robot‐assisted Roux‐en‐Y gastric bypass; TRRYGB, totally robot‐assisted Roux‐en‐Y gastric bypass; RPD, robot‐assisted pancreatoduodenectomy; RARP, robot‐assisted radical prostatectomy; ORP, open radical prostatectomy; HAI, hepatic artery infusion; REVUR, robot‐assisted extravesical ureteral reimplantation; RATS, robot‐assisted thoracic surgery; RSC, robot‐assisted sacrocolpopexy; LRP, laparoscopic radical prostatectomy; RAT, robot‐assisted thymectomy; RRS, robot‐assisted rectal cancer surgery; LESS, laparoendoscopic single‐site; RC, robot‐assisted cholecystectomy; TME, total mesorectal excision; RAG, robot‐assisted gastrectomy; RAPN, robot‐assisted partial nephrectomy; SSRC, single‐site robot‐assisted cholecystectomy; LSC, laparoscopic sacrocolpopexy; RALP, robot‐assisted laparoscopic prostatectomy; RA‐GPEHR, robot‐assisted giant para‐oesophageal hernia repair; RKT, robot‐assisted kidney transplantation; OKT, open kidney transplantation; LND, lymph node dissection.

### Learning curve metrics

**Figure 4:**
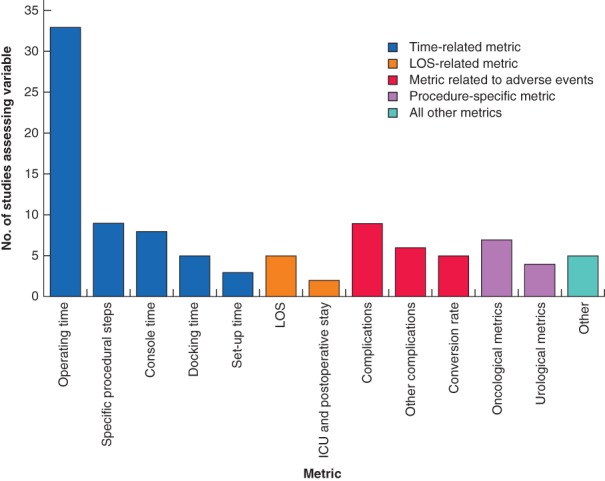
Bar chart of the learning curve metrics assessed across the captured studies LOS, length of stay.

Time‐based metrics were the most commonly reported variables used to assess the learning curve, reported by 42 of the 49 studies (86 per cent). Other measures, including length of hospital stay, morbidity and mortality rates, and procedure‐specific metrics, were reported less commonly (*Fig*. 4). Of the categories of metrics captured, duration of surgery, length of stay (LOS) and complication rate were reported most frequently within each category. The number of procedures required to overcome the learning curve for these metrics is shown in *Table* 2.

**Table 2:** Learning curve results for duration of surgery, length of stay and complication rate

Metric	Procedure	No. of robotic surgeons	Procedures to overcome learning curve (subgroup)*	Specific threshold in surgeon performance	Reference
**Operating time**	**Urology**				
	RARP	2	> 15 (expert laparoscopic surgeon)	No. of procedures to reach break point	Lebeau *et al*.^30^
			15 (junior surgeon)		
		3	140 (open experience)	No. of procedures to reach plateau	Chang *et al*.^16^
			40 (open/laparoscopic experience)		
			> 79 (laparoscopic experience)		
	Robot‐assisted pyeloplasty	4	37 (fellows)	No. of procedures to achieve median operating time of an attending surgeon	Tasian *et al*.^50^
	Robot‐assisted adrenalectomy	n.r.	12	No. of procedures to reach plateau	D'Annibale *et al*.^18^
	RAPN	3	> 100	Identifying a plateau effect	Tobis *et al*.^51^
	RALP	2	20	No. of procedures to approach median operative duration of LRP and presence of a plateau effect	Wolanski *et al*.^56^
	**General surgery**				
	Robot‐assisted HAI pump placement	n.r.	8	No. of procedures to reach a decline in CUSUM curve	Dhir *et al*.^20^
	RPD	4	80	No. of procedures to reach a decline in CUSUM curve	Boone *et al*.^15^
		n.r.	> 132	n.r.	Zureikat *et al*.^58^
	Robot‐assisted thyroidectomy	2	19; 20	No. of procedures to reach plateau	Park *et al*.^39^
	RDP	2	> 11	n.r.	Benizri *et al*.^12^
		3	40	No. of procedures to reach a decline in CUSUM curve	Shakir *et al*.^47^
		n.r.	> 83	n.r.	Zureikat *et al*.^58^
	RAG	3	8·2	No. of procedures to reach stabilization	Park *et al*.^40^
		2	14; 21	No. of procedures to reach a decline in CUSUM curve	Zhou *et al*.^57^
	RC	8	> 6	n.r.	Nelson *et al*.^37^
	SSRC	5	0	n.r.	Pietrabissa *et al*.^42^
	**Gynaecology**				
	RSC	2	> 24; 60	No. of procedures to reach plateau	Linder *et al*.^31^
		3	0	Moving block technique to detect a significant drop‐off	Pulliam *et al*.^43^
	Robot‐assisted hysterectomy	1315	> 150	n.r.	Luciano *et al*.^34^
	**Colorectal**				
	Robot‐assisted colorectal resection	4	74 (early adapter)	No. of procedures to reach a CUSUM curve phase change	Guend *et al*.^27^
			25–30 (later adapters)		
	Robot‐assisted TME	2	7; 15	No. of procedures to achieve comparable performance to laparoscopy via CUSUM analysis	Odermatt *et al*.^38^
	RRS	2	17 (laparoscopically inexperienced)	No. of procedures to reach plateau	Kim *et al*.^29^
			0 (laparoscopically experienced)		
	**Thoracic**				
	RAT	4	15–20	No. of procedures to reach plateau	Kamel *et al*.^28^
	Robot‐assisted lobectomy	2	15	No. of procedures to reach plateau	Meyer *et al*.^35^
	**Paediatric**				
	Laparoscopic robot‐assisted fundoplications	2	25	No. of procedures to reach plateau	Binet *et al*.^14^
	REVUR	4	7–8	No. of procedures to reach plateau	Esposito *et al*.^21^
	**Cardiovascular**				
	Robot‐assisted mitral valve repair	2	> 404	No. of procedures to reach plateau	Goodman *et al*.^26^
**Length of stay**	**Colorectal**				
	Robot‐assisted TME	2	0; 15	No. of procedures to achieve comparable performance to laparoscopy via CUSUM analysis	Odermatt *et al*.^38^
	**Thoracic**				
	Robot‐assisted lobectomy	2	> 185	No. of procedures to reach plateau	Meyer *et al*.^35^
**Complications**	**Urology**				
	LND during RALP	2	> 400	No. of procedures to reach plateau	van der Poel *et al*.^52^
	**General surgery**				
	Robot‐assisted pancreatic resections	n.r.	> 132	n.r.	Zureikat *et al*.^58^
	**Gynaecology**				
	RSC	2	0	Proficiency set at less than 10% complication rate	Myers *et al*.^36^
		2	> 24; 84	Proficiency defined as point where CUSUM curve crossed and consistently stayed below reference line of 0 (based on expected complication rate)	Linder *et al*.^31^
	Robot‐assisted hysterectomy	2	12; 14	Proficiency defined as the point where CUSUM curve crossed lower control limit H_0_	Woelk *et al*.^55^
		1315	> 150	n.r.	Luciano *et al*.^34^
	**Colorectal**				
	Robot‐assisted TME	2	0; 15	No. of procedures to achieve comparable performance to laparoscopy via CUSUM analysis	Odermatt *et al*.^38^
	**Cardiovascular**				
	Robot‐assisted mitral valve repair	2	> 404	No. of procedures to reach plateau	Goodman *et al*.^26^

Studies that did not report whether the learning curve had or had not been overcome within the study period were not included in this table.

* For studies that reported a consistent improvement in metrics across the course of the study, it was assumed that the learning curve had not been overcome within the study period. If the learning curve had not been overcome, the number of procedures to overcome the learning curve was reported to be greater than (>) the total number of procedures in the study period. If the learning curve was reported to have been overcome (or surgeons were reported to be proficient/competent) before study initiation, the number of procedures required to overcome the learning curve was recorded as zero. Where results were reported separately for individual surgeons with no clear differences in previous experience, learning curve estimates are reported separately, separated by a semicolon; where the experience of surgeons was intentionally different, individual experience is reported as separate rows and experience level is stated in brackets. RARP, robot‐assisted radical prostatectomy; n.r., not reported; RAPN, robot‐assisted partial nephrectomy; RALP, robot‐assisted laparoscopic prostatectomy; LRP, laparoscopic radical prostatectomy; HAI, hepatic artery infusion; CUSUM, cumulative sum; RPD, robot‐assisted pancreatoduodenectomy; RDP, robot‐assisted distal pancreatectomy; RAG, robot‐assisted gastrectomy; RC, robot‐assisted cholecystectomy; SSRC, single‐site robot‐assisted cholecystectomy; RSC, robot‐assisted sacrocolpopexy; TME: total mesorectal excision; RRS, robot‐assisted rectal cancer surgery; RAT, robot‐assisted thymectomy; REVUR, robot‐assisted extravesical ureteral reimplantation; LND, lymph node dissection.

#### Duration of surgery

Across 33 studies that investigated the learning curve based on duration of surgery, 27 reported whether the learning curve had been overcome. In 21 of these 27 studies (78 per cent), at least one of the included surgeons was reported to have overcome the learning curve, with the remaining six studies (22 per cent) stating that the learning curve had not been overcome by any surgeon within the number of procedures in the study period (*Table* 2).

Among studies where the learning curve was not overcome by any surgeon, the number of procedures assessed over the study period varied significantly, with a range of 6–404 patients^12^, ^26^, ^34^, ^37^, ^51^, ^58^. Of the studies in which the learning curve had been overcome, 12–140 patients were needed for urological procedures^16^, ^18^, ^30^, ^50^, ^56^, 0–80 for general surgical procedures (where 0 suggests no apparent learning process required, if the surgeon was already experienced)^15^, ^20^, ^39^, ^40^, ^42^, ^47^, ^57^, 0–60 for gynaecological procedures^31^, ^43^, 0–74 for colorectal procedures^27^, ^29^, ^38^, 15–20 for thoracic procedures^28^, ^35^ and 7–25 for paediatric procedures^14^, ^21^.

Among the 27 studies that reported whether the learning curve had been overcome, learning curve analyses were conducted for 22 unique procedures, of which only five (23 per cent) were supported by multiple studies. In such instances, the number of patients required to overcome the learning curve for these varied substantially between studies. For example, three studies evaluated the learning curve for duration of surgery for robot‐assisted distal pancreatectomy. In one study^47^ the learning curve was overcome after 40 patients, whereas in the other two^12^, ^58^ it had not been overcome within the study period (of 11 and 83 patients).

#### Length of stay

Of five studies reporting LOS, two reported on whether the learning curve had been overcome by at least one robotic surgeon (*Table* 2). One study^38^ estimated that the number of patients required to overcome the learning curve was between 0 and 15. In the other study^35^, the learning curve was not overcome within a study period of 185 patients.

#### Complications

Of nine studies assessing complications, eight reported whether the learning curve was overcome for complication rate. Of these, five^26^, ^31^, ^34^, ^52^, ^58^ found that the learning curve for complications had not been overcome for at least one robotic surgeon within the study period. With the exception of one study^31^, which included a surgeon with a study period of only 24 patients, these studies generally involved large patient numbers (132–404), and spanned urology, general surgery, gynaecology and cardiovascular specialties (*Table* 2). Only four studies reported that the learning curve for complications had been overcome. The numbers of procedures were estimated as: 0–84 for robot‐assisted sacrocolpopexy^31^, ^36^, 12–14 for robot‐assisted hysterectomy^55^ and 0–15 for robot‐assisted total mesorectal excision^38^.

#### Clinical metrics

Of the 49 studies, eight (16 per cent) evaluated whether the learning curve for clinical metrics had been overcome (*Table* 3). Metrics included oncology‐specific metrics such as surgical margin status and recurrence rate, and urology‐specific metrics such as urinary continence. The number of procedures to overcome the learning curve varied substantially. Of the two studies assessing urinary continence after robot‐assisted radical prostatectomy, one^25^ reported that 100 procedures were required to overcome the learning curve, whereas in the other^23^ the learning curve was not overcome by any of the four robotic surgeons, with study periods ranging from 112 to 541 patients. For all other clinical metrics, the learning curve was overcome by at least one surgeon during the study period, with a wide range of 0–300 patients to achieve this target.

**Table 3:** Learning curve results for clinical metrics

Metric group	Specific metric	Procedure	No. of robotic surgeons	Procedures to overcome learning curve (subgroup)*	Specific threshold in surgeon performance	Reference
Urinary continence	Probability of UC recovery after 1 year	RARP	4	> 112; > 411; > 413; > 541	No. of procedures to reach plateau	Fossati *et al*.^23^
	Early UC	RARP	1	100	No. of procedures to reach plateau	Good *et al*.^25^
Renal function	Serum creatinine level	RKT	3	0 (extensive RKT, limited OKT)	No. of procedures to reach ‘transition point’ in CUSUM curve	Sood *et al*.^49^
				0 (extensive RKT/OKT)		
				3 (limited RKT, extensive OKT)		
	Glomerular filtration rate	RKT	3	0 (extensive RKT, limited OKT)	No. of procedures to reach ‘transition point’ in CUSUM curve	Sood *et al*.^49^
				0 (extensive RKT/OKT)		
				3 (limited RKT, extensive OKT)		
Biochemical recurrence	Biochemical recurrence	RARP	9	100	No. of procedures to reach ‘transition point’ in CUSUM curve	Sivaraman *et al*.^48^
Surgical margins	Positive surgical margins	RARP	9	100	No. of procedures to reach ‘transition point’ in CUSUM curve	Sivaraman *et al*.^48^
	Apical positive surgical margins	RARP	1	0	No. of procedures to reach plateau	Good *et al*.^25^
	Node positivity rate	LND during RALP	2	300	No. of procedures to reach plateau	van der Poel *et al*.^52^
	Initial margin status	TORS	3	15; 22; > 68	No. of procedures to reach inflection point in CUSUM curve	Albergotti *et al*.^10^
	Final positive surgical margins	TORS	3	25; 27; > 37	No. of procedures to reach inflection point in CUSUM curve	Albergotti *et al*.^10^
Lymph node yield	No. of removed nodes	LND during RALP	2	250	No. of procedures to reach plateau	van der Poel *et al*.^52^
	Lymph node harvest	RPD	4	80	No. of procedures to reach significant improvement	Boone *et al*.^15^
	Lymph node harvest	Robot‐assisted TME	2	0	No. of procedures to achieve comparable performance to laparoscopy via CUSUM analysis	Odermatt *et al*.^38^

Studies that did not report whether the learning curve had or had not been overcome within the study period are not included in this table.

* For studies that reported a consistent improvement in metrics across the course of the study, it was assumed that the learning curve had not been overcome within the study period. If the learning curve had not been overcome, the number of procedures to overcome the learning curve was reported to be greater than (>) the total number of procedures in the study period. If the learning curve was reported to have been overcome (or surgeons were reported to be proficient/competent) before study initiation, the number of procedures to overcome the learning curve was recorded as zero. Where results were reported separately for individual surgeons with no clear differences in previous experience, learning curve estimates are reported separately, separated by a semicolon; where the experience of surgeons was intentionally different, individual experience is reported as separate rows and experience level is stated in brackets. UC, urinary continence; RARP, robot‐assisted radical prostatectomy; RKT, robot‐assisted kidney transplantation; OKT, open kidney transplantation; CUSUM, cumulative sum; LND, lymph node dissection; RALP, robot‐assisted laparoscopic prostatectomy; TORS, transoral robotic surgery; RPD, robot‐assisted pancreatoduodenectomy; TME, total mesorectal excision.

#### Within‐study comparison between metrics

Some studies in the review evaluated the learning curve using more than one metric. The number of patients to overcome the learning curve was sometimes inconsistent between metrics. For example, of the two studies^35^, ^38^ that reported whether or not the learning curve was overcome for both duration of surgery and LOS, one^35^ indicated that substantially greater procedural experience was required for LOS, with more than 170 additional patients required to overcome the learning curve based on this metric.

### Standards of reporting

The overall standard of reporting and level of detail provided in the included studies was low, often lacking sufficient information to interpret the learning curve for robot‐assisted procedures. For example, although 34 of the 49 studies (69 per cent) made some acknowledgement relating to the previous experience of included surgeons (*Table* 4), the detail in the reporting was mixed, with only 17 of 49 studies (35 per cent) quantifying previous experience of robot‐assisted (8 of 49, 16 per cent), laparoscopic (13 of 49, 27 per cent) or open (6 of 49, 12 per cent) operations completed. Only four of 49 studies (8 per cent) indicated whether simulation, dry lab or cadaver training had been completed before patient enrolment.

**Table 4:** Studies reporting previous surgeon experience

	Surgeon's previous procedure experience				
Reference	Robot‐assisted	Laparoscopic	Open	Approach not defined	Simulated robotic training before study
Albergotti *et al*.^10^	✘	✘	✘	**?**	✘
Arora *et al*.^11^	**?**	✘	✘	**?**	✘
Benizri *et al*.^12^	✘	✘	✘	✘	**?**
Bindal *et al*.^13^	✘	**?**	✘	✘	✘
Binet *et al*.^14^	✘	**?**	✘	✘	✘
Chang *et al*.^16^	✘	**?**	**?**	✘	✘
Dhir *et al*.^20^	✘	✘	**?**	✘	✘
Geller *et al*.^24^	**✓**	✘	✘	**?**	**?**
Good *et al*.^25^	**✓**	**✓**	**✓**	✘	**?**
Goodman *et al*.^26^	**✓**	✘	**?**	✘	**✓**
Guend *et al*.^27^	**?**	**?**	✘	✘	✘
Kamel *et al*.^28^	✘	**?**	✘	✘	✘
Kim *et al*.^29^	✘	**✓**	**✓**	✘	**?**
Lebeau *et al*.^30^	✘	**✓**	**✓**	✘	✘
Lopez *et al*.^32^	**✓**	**✓**	✘	✘	✘
Lovegrove *et al*.^33^	**?**	✘	✘	✘	✘
Meyer *et al*.^35^	✘	**✓**	✘	✘	✘
Odermatt *et al*.^38^	✘	**✓**	✘	✘	**✓**
Park *et al*.^40^	✘	**✓**	✘	✘	✘
Park *et al*.^39^	**✓**	**✓**	**✓**	✘	✘
Pietrabissa *et al*.^42^	**✓**	✘	✘	✘	**✓**
Pulliam *et al*.^43^	**?**	**?**	✘	✘	✘
Riikonen *et al*.^44^	✘	**?**	**?**	✘	✘
Sarkaria *et al*.^45^	✘	**?**	✘	✘	✘
Shakir *et al*.^47^	**?**	**?**	✘	✘	✘
Sivaraman *et al*.^48^	✘	**✓**	✘	✘	✘
Sood *et al*.^49^	**✓**	✘	**✓**	✘	✘
Tasian *et al*.^50^	**?**	✘	✘	✘	✘
Tobis *et al*.^51^	**✓**	**✓**	✘	✘	✘
van der Poel *et al*.^52^	✘	**✓**	**✓**	✘	**?**
Woelk *et al*.^55^	**?**	✘	✘	**?**	✘
Wolanski *et al*.^56^	✘	**✓**	**?**	✘	**✓**
Zhou *et al*.^57^	✘	**✓**	✘	✘	✘
Zureikat *et al*.^58^	✘	✘	✘	**?**	✘

**✓**, Study quantified the amount of previous experience (procedures, days of simulated training); ?, study acknowledged previous experience but did not quantify it; ✘, study did not acknowledge previous experience.

**Figure 5:**
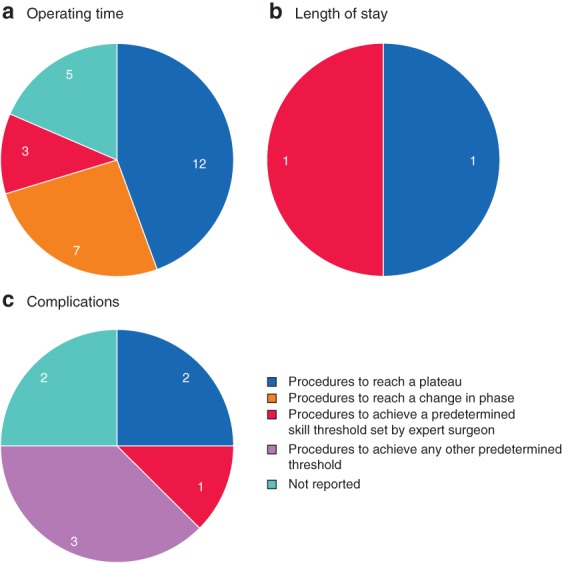
Pie chart of methods used to define the point at which learning curve was overcome **a** Operating time; **b** length of stay; **c** complications. Values indicate the number of studies using the method.

Variability was observed in the performance thresholds used to measure the learning curve. For example, among the 27 studies that defined whether the learning curve for duration of surgery had been overcome (*Table* 2), the most common performance threshold used was the number of procedures needed to reach a plateau in performance, but other thresholds included the number of procedures to reach a change in phase, or the number to achieve a predetermined skill threshold set by an expert surgeon, and some studies did not specify the performance thresholds used (*Fig*. 5 *a*). In nine of the 27 studies (33 per cent), no statistical or quantitative assessment of the learning curve was reported beyond visual fit or a qualitative description (data not reported). Similar variation in learning curve definitions was observed for analyses of LOS and complication rates (*Fig*. 5 *b,c*).

Several studies used these methods to define whether surgeons had achieved a high level of performance, characterized by terms such as proficiency or competency. However, these terms were used inconsistently. In 13 of 14 studies (93 per cent) that employed performance terms, at least one term was used to describe the point where the learning curve had been overcome; ‘proficiency’ was used for this purpose in ten studies^22^, ^27^, ^28^, ^31^, ^36^, ^38^, ^39^, ^42^, ^50^, ^55^, ‘competency’ in five studies^10^, ^27^, ^33^, ^36^, ^49^ and ‘expertise’ in one study^50^. In the four studies that reported more than one performance term, the terms were either used interchangeably^36^, ^50^ or assigned divergent definitions^33^, ^49^.

Although quality assessment of included studies (*Table* *S5*, supporting information) revealed a relatively low risk of bias for several quality assessment items, risk of bias was unclear for a large proportion of the questions, suggesting poor reporting of methodology. In particular, the risk of bias with respect to the blinding of subjects, external validity of included populations and study centres, and statistical power was either high or could not be determined (at least 45 of the 49 studies, 92 per cent).

## Discussion

This review identified substantial variation in the lengths of learning curves, included metrics and methods employed to assess the learning curve, as well as the reporting of the analyses and terminology used across ten surgical specialties. Reported learning curve estimates are therefore subject to substantial uncertainty, and the generalizability of these findings is limited.

The results of the 49 eligible studies suggested that surgeon learning curves were complex. They varied significantly between studies, procedures and specific metrics assessed. A variety of factors could account for much of the variation in reported learning curve length.

The surgeon's previous experience may have been a significant factor; in three of five studies comparing the operating time learning curves of robotic surgeons, those with greater experience required fewer procedures to overcome their learning curve^16^, ^29^, ^38^. Although the captured studies often compared surgeons with different experience levels, such as trainees *versus* those who had completed training or robotic *versus* laparoscopic surgeons, studies generally did not report the participants' specific grade or training experience.

Robotic training programmes are becoming increasingly common to enable surgeons to overcome the learning curve faster^59^, ^60^. In addition to previous experience, participation in specific training programmes may influence the learning process. Although based on a small sample size, Guend and colleagues^27^ reported that a lower procedure volume was required to overcome the learning curve for robot‐assisted colorectal resections for three surgeons who had participated in an institutional training programme (25–30 procedures each), compared with the volume required for an earlier surgeon who joined the institution before the programme was established (74 procedures). Few studies, however, provided details of their training programmes, where these existed. Recent training programmes have considered innovations such as feedback loops that aim to provide specific recommendations for improvement, shortening the time required to achieve adequate performance^61^.

Differences in procedural complexity may also have contributed to variation in the learning curves observed. In surgical practice, following initial improvement and subsequent stabilization of performance, a decline in performance is often observed^62^. This decline is thought to reflect the point at which, following mastery of simpler procedures, surgeons take on more challenging, technically complex procedures, that impact on a learning curve^63^.

Some procedures are inherently more complex and challenging than others. Studies^64^, ^65^, ^66^ of simulated robotic training tasks have observed learning curves of different duration for tasks of varying complexity. Learning curves are likely to be influenced by numerous observed and unobserved confounders. To account better for such differences, and to permit comparisons between studies, enhanced reporting of surgeon baseline characteristics, experience and procedure complexity is required.

This review has highlighted a number of limitations associated with reporting learning curves for robot‐assisted surgery. Many studies failed to describe the characteristics of the surgeons, patients or methods of assessment in sufficient detail to make valid comparisons between studies or to enable a study to be reproduced.

Although the majority of captured studies were determined to be of reasonable methodological quality, the studies were observational and usually included few surgeons. These study designs are associated with significant drawbacks, particularly with respect to confounding and selection bias^67^, ^68^, although this is expected given that they are more suited to measuring learning curves. Regarding sources of bias in the included studies, the risk of bias was unclear for a large proportion of the quality assessment items, particularly in relation to blinding, external validity and statistical power, suggesting poor reporting of methodology.

There was little consistency in the performance thresholds used to measure the learning curve, making between‐study comparisons challenging. A large proportion of studies measured the number of procedures required to reach a plateau in surgeon performance. Although a variety of methods can define quantitatively the point at which a plateau is reached^69^, ^70^, ^71^, ^72^, there is currently no widely accepted and validated method, and some studies used visual fit alone^1^. The number of procedures required to overcome learning curves reported are subject to considerable uncertainty. Several studies measured the number of procedures to achieve a threshold set by experts. These were sometimes based on the performance of expert robotic surgeons^50^, whereas others^38^, ^56^ included expert laparoscopic surgeons. Many studies did not report the specific performance thresholds used to measure the learning curve, precluding any ability to make comparisons.

These methods were frequently used to define the points at which surgeons reached ‘proficiency’, ‘competency’ or other related terms. These terms, however, were used inconsistently or interchangeably^36^, ^50^, or used with distinct definitions^33^, ^49^. In one study^49^ competency was used to describe performance that reached a steady state or plateau, whereas proficiency described further improvement after plateau and mastery as the achievement of outcomes better than the set target value. These definitions are not well aligned with guidelines for assessing surgical competence^73^, ^74^, recommended by the US Accreditation Council for Graduate Medical Education and the American Board of Medical Specialties, nor the criteria developed for procedure‐based assessment in the Intercollegiate Surgical Curriculum Programme in the UK.

The mismatch between the performance thresholds used to measure the learning curve and the terminology used to describe the results of the analyses can lead to misinterpretation. The term ‘overcome’ was commonly used to describe a point when surgeons reached a given performance threshold, implying a high level of performance. These thresholds, particularly time to plateau, are often simplistic and may not capture sufficient evidence about the learning process to support this implication. A plateau in performance does not always equate with high‐quality performance, as surgeons will not necessarily plateau at the same level^1^. Likewise, using thresholds of performance to define terms such as competence and proficiency could be considered inappropriate, as these terms also imply a specific level of performance. A recent study^61^ investigated proficiency‐based progression training programmes in which residents who failed to show progressive improvement (reached a plateau in performance) were not considered proficient unless they had achieved predetermined proficiency benchmarks set by experienced surgeons.

The lack of consistency in methods used to describe surgical performance and the use of simplistic and inappropriate methods adds to the complexity of interpreting learning curves. Using thresholds that provide meaningful measures of surgeon performance alongside standardized terminology seems vital to realize the full potential of learning curve analyses for optimization of surgical training programmes.

Time‐based variables were the most common metrics used to assess learning curves, as is the case for other systematic reviews assessing surgical learning curves in other contexts^63^, ^75^. Although common across learning curve analyses, the present review suggests that variation can exist between the learning curve profiles of different metrics for a given procedure, with recovery and safety metrics (LOS, complications) exhibiting substantially longer learning curves than those for operating time, often with continued improvement for extended periods of time after the learning curve for operating time has been overcome. Given that improvements in clinical outcomes may be important drivers for the uptake of robot‐assisted approaches, the value of comparisons based on learning curves for operating time alone is unclear. In addition, the metrics captured may not directly measure surgical performance, with surrogate markers, such as operating time and LOS, reported frequently. Real‐time automated performance metrics, coupled with machine learning algorithms to process automatically collected data, may enable more direct measurement of surgeon performance^76^.

Several data gaps were identified in the reported data. Many of the identified studies enrolled fully trained surgeons, investigating the transferability of skills for conversion from laparoscopic or open surgery to robot‐assisted procedures. These studies may be of limited value for informing the design of surgical training programmes. The limited data investigating the learning curves of trainee surgeons may result in missed opportunities for the optimization of programmes to accelerate the training of surgeons who are novices with robot‐assisted devices. No study reported data related to the economic impact of the learning curve, such as training costs or financial impacts of suboptimal outcomes.

This systematic review was a broad, exploratory search of the literature reporting on the surgeon learning curve for robot‐assisted surgery, with broad search terms and eligibility criteria. Incomplete and variable reporting created challenges for data synthesis, especially given some of the exploratory and subjective outcomes this review intended to identify. The exploratory nature of the review may have introduced a number of limitations, which may have resulted in relevant data being overlooked. For example, to include evidence of suitable quality, studies that involved fewer than 20 surgical procedures in total (across surgical approaches or surgeons) were excluded, regardless of the number of robot‐assisted procedures completed by any one surgeon or included within the learning curve analysis. Studies were captured only if they reported actual learning curve data (as a graph or table presenting at least 4 time points), so that studies reporting potentially relevant data (for example the economic impact of the learning curve) could have been excluded if they did not meet these criteria. Studies that included just a single surgeon were also excluded, as the innate differences in technical ability that may exist between surgeons were anticipated to limit the reliability of the data reported in these studies. Only the learning curves of robotic procedures were considered in this review, and although the review did not set out to compare robotic‐assisted procedures with other surgical approaches, this prevented any conclusions to be drawn regarding lengths of the learning curve for robot‐assisted *versus* non‐robotic procedures. Only studies originally published in English were included, and database searches were limited to 2012–2018 in order to capture evidence most relevant to present‐day training practices. Although comparisons between robot‐assisted and other surgical approaches are warranted, studies with appropriate evaluation methods, standardized terminology and necessary context are essential for robust comparisons to be made. These kinds of study should provide better estimates of learning curves for robot‐assisted procedures, enhance surgical training programmes and improve patient outcomes.

## Supporting information


**Table S1.** Search terms for MEDLINE, MEDLINE In‐Process, MEDLINE Epub Ahead of Print and Embase
**Table S2.** Search terms for Cochrane Library databases (searched via Wiley Online platform)
**Table S3.** Full eligibility criteria
**Table S4.** List of studies excluded at full‐text review and reasons for exclusion
**Table S5.** Quality assessment of non‐randomized articles using Downs and Black checklist

## References

[1] Khan N , Abboudi H , Khan MS , Dasgupta P , Ahmed K . Measuring the surgical ‘learning curve’: methods, variables and competency. BJU Int 2014; 113: 504–508. 23819461 10.1111/bju.12197

[2] Randell R , Alvarado N , Honey S , Greenhalgh J , Gardner P , Gill A . Impact of robotic surgery on decision making: perspectives of surgical teams. AMIA Annu Symp Proc 2015; 2015: 1057–1066. 26958244 PMC4765621

[3] Lanfranco AR , Castellanos AE , Desai JP , Meyers WC . Robotic surgery: a current perspective. Ann Surg 2004; 239: 14. 14685095 10.1097/01.sla.0000103020.19595.7d PMC1356187

[4] Cundy TP , Harling L , Hughes‐Hallett A , Mayer EK , Najmaldin AS , Athanasiou T *et al* Meta‐analysis of robot‐assisted *vs* conventional laparoscopic and open pyeloplasty in children. BJU Int 2014; 114: 582–594. 25383399 10.1111/bju.12683

[5] Agrusa A , Romano G , Navarra G , Conzo G , Pantuso G , Buono GD *et al* Innovation in endocrine surgery: robotic *versus* laparoscopic adrenalectomy. Meta‐analysis and systematic literature review. Oncotarget 2017; 8: 102 392–102 400. 10.18632/oncotarget.22059 PMC5731964 29254254

[6] Turchetti G , Palla I , Pierotti F , Cuschieri A . Economic evaluation of da Vinci‐assisted robotic surgery: a systematic review. Surg Endosc 2012; 26: 598–606. 21993935 10.1007/s00464-011-1936-2

[7] Moher D , Liberati A , Tetzlaff J , Altman DG ; PRISMA Group . Preferred reporting items for systematic reviews and meta‐analyses: the PRISMA statement. BMJ 2009; 339: b2535. 19622551 10.1136/bmj.b2535 PMC2714657

[8] National Institute for Health and Care Excellence (NICE) . Single Technology Appraisal: User Guide for Company Evidence Submission Template. NICE: London, 2015.

[9] Downs SH , Black N. The feasibility of creating a checklist for the assessment of the methodological quality both of randomised and non‐randomised studies of health care interventions. J Epidemiol Community Health 1998; 52: 377–384. 9764259 10.1136/jech.52.6.377 PMC1756728

[10] Albergotti WG , Gooding WE , Kubik MW , Geltzeiler M , Kim S , Duvvuri U *et al* Assessment of surgical learning curves in transoral robotic surgery for squamous cell carcinoma of the oropharynx. JAMA Otolaryngol Head Neck Surg 2017; 143: 542–548. 28196200 10.1001/jamaoto.2016.4132 PMC5614443

[11] Arora S , Tugcu V , Sood A , Bhandari M , Ahlawat R , Menon M . Learning curve of a new surgical procedure: experience from a new center adopting robotic kidney transplant. J Urol 2017; 197: e73–e74.

[12] Benizri EI , Germain A , Ayav A , Bernard JL , Zarnegar R , Benchimol D *et al* Short‐term perioperative outcomes after robot‐assisted and laparoscopic distal pancreatectomy. J Robot Surg 2014; 8: 125–132. 27637522 10.1007/s11701-013-0438-8

[13] Bindal V , Gonzalez‐Heredia R , Masrur M , Elli EF . Technique evolution, learning curve, and outcomes of 200 robot‐assisted gastric bypass procedures: a 5‐year experience. Obes Surg 2015; 25: 997–1002. 25394589 10.1007/s11695-014-1502-9

[14] Binet A , Fourcade L , Amar S , Alzahrani K , Cook AR , Braïk K *et al* Robot‐assisted laparoscopic fundoplications in pediatric surgery: experience review. Eur J Pediatr Surg 2019; 29: 173–178. 29258148 10.1055/s-0037-1615279

[15] Boone BA , Zenati M , Hogg ME , Steve J , Moser AJ , Bartlett DL *et al* Assessment of quality outcomes for robotic pancreaticoduodenectomy: identification of the learning curve. JAMA 2015; 150: 416–422. 10.1001/jamasurg.2015.17 25761143

[16] Chang Y , Qu M , Wang L , Yang B , Chen R , Zhu F *et al* Robotic‐assisted laparoscopic radical prostatectomy from a single Chinese center: a learning curve analysis. Urology 2016; 93: 104–111. 27045710 10.1016/j.urology.2016.03.036

[17] Ciabatti PG , Burali G , D'Ascanio L . Single‐incision robot‐assisted transaxillary surgery for early‐stage papillary thyroid cancer. Ann Otol Rhinol Laryngol 2012; 121: 811–815. 23342554 10.1177/000348941212101207

[18] D'Annibale A , Lucandri G , Monsellato I , De Angelis M , Pernazza G , Alfano G *et al* Robotic adrenalectomy: technical aspects, early results and learning curve. Int J Med Robot 2012; 8: 483–490. 23081692 10.1002/rcs.1454

[19] Davis JW , Kreaden US , Gabbert J , Thomas R . Learning curve assessment of robot‐assisted radical prostatectomy compared with open‐surgery controls from the premier perspective database. J Endourol 2014; 28: 560–566. 24350787 10.1089/end.2013.0534 PMC3995359

[20] Dhir M , Zenati MS , Padussis JC , Jones HL , Perkins S , Clifford AK *et al* Robotic assisted placement of hepatic artery infusion pump is a safe and feasible approach. J Surg Oncol 2016; 114: 342–347. 27529576 10.1002/jso.24325

[21] Esposito C , Masieri L , Steyaert H , Escolino M , Cerchione R , La Manna A *et al* Robot‐assisted extravesical ureteral reimplantation (REVUR) for unilateral vesico‐ureteral reflux in children: results of a multicentric international survey. World J Urol 2018; 36: 481–488. 29248949 10.1007/s00345-017-2155-9

[22] Fahim C , Hanna W , Waddell T , Shargall Y , Yasufuku K . Robotic‐assisted thoracoscopic surgery for lung resection: the first Canadian series. Can J Surg 2017; 60: 260–265. 28562237 10.1503/cjs.005316 PMC5529157

[23] Fossati N , Di Trapani E , Gandaglia G , Dell'Oglio P , Umari P , Buffi NM *et al* Assessing the impact of surgeon experience on urinary continence recovery after robot‐assisted radical prostatectomy: results of four high‐volume surgeons. J Endourol 2017; 31: 872–877. 28732186 10.1089/end.2017.0085

[24] Geller EJ , Lin FC , Matthews CA . Analysis of robotic performance times to improve operative efficiency. J Minim Invasive Gynecol 2013; 20: 43–48. 23141423 10.1016/j.jmig.2012.08.774

[25] Good DW , Stewart GD , Laird A , Stolzenburg JU , Cahill D , McNeill SA . A critical analysis of the learning curve and postlearning curve outcomes of two experience‐ and volume‐matched surgeons for laparoscopic and robot‐assisted radical prostatectomy. J Endourol 2015; 29: 939–947. 25630790 10.1089/end.2014.0810

[26] Goodman A , Koprivanac M , Kelava M , Mick SL , Gillinov AM , Rajeswaran J *et al* Robotic mitral valve repair: the learning curve. Innovations 2017; 12: 390–397. 29232301 10.1097/IMI.0000000000000438

[27] Guend H , Widmar M , Patel S , Nash GM , Paty PB , Guillem JG *et al* Developing a robotic colorectal cancer surgery program: understanding institutional and individual learning curves. Surg Endosc 2017; 31: 2820–2828. 27815742 10.1007/s00464-016-5292-0 PMC5418100

[28] Kamel MK , Rahouma M , Stiles BM , Nasar A , Altorki NK , Port JL . Robotic thymectomy: learning curve and associated perioperative outcomes. J Laparoendosc Adv Surg Tech A 2017; 27: 685–690. 28121481 10.1089/lap.2016.0553

[29] Kim IK , Kang J , Park YA , Kim NK , Sohn SK , Lee KY . Is prior laparoscopy experience required for adaptation to robotic rectal surgery?: feasibility of one‐step transition from open to robotic surgery. Int J Colorectal Dis 2014; 29: 693‐699. 24770702 10.1007/s00384-014-1858-2

[30] Lebeau T , Rouprêt M , Ferhi K , Chartier‐Kastler E , Bitker MO , Richard F *et al* The role of a well‐trained team on the early learning curve of robot‐assisted laparoscopic procedures: the example of radical prostatectomy. Int J Med Robot 2012; 8: 67–72. 22556136 10.1002/rcs.435

[31] Linder BJ , Anand M , Weaver AL , Woelk JL , Klingele CJ , Trabuco EC *et al* Assessing the learning curve of robotic sacrocolpopexy. Int Urogynecol J 2016; 27: 239–246. 26294206 10.1007/s00192-015-2816-4

[32] Lopez S , Mulla ZD , Hernandez L , Garza DM , Payne TN , Farnam RW . A comparison of outcomes between robotic‐assisted, single‐site laparoscopy versus laparoendoscopic single site for benign hysterectomy. J Minim Invasive Gynecol 2016; 23: 84–88. 26321172 10.1016/j.jmig.2015.08.883

[33] Lovegrove C , Novara G , Mottrie A , Guru KA , Brown M , Challacombe B *et al* Structured and modular training pathway for robot‐assisted radical prostatectomy (RARP): validation of the RARP assessment score and learning curve assessment. Eur Urol 2016; 69: 526–535. 26585582 10.1016/j.eururo.2015.10.048

[34] Luciano AA , Luciano DE , Gabbert J , Seshadri‐Kreaden U . The impact of robotics on the mode of benign hysterectomy and clinical outcomes. Int J Med Robot 2016; 12: 114–124. 25753111 10.1002/rcs.1648

[35] Meyer M , Gharagozloo F , Tempesta B , Margolis M , Strother E , Christenson D . The learning curve of robotic lobectomy. Int J Med Robot 2012; 8: 448–452. 22991294 10.1002/rcs.1455

[36] Myers EM , Geller EJ , Connolly A , Bowling JM , Matthews CA . Robotic sacrocolpopexy performance and cumulative summation analysis. Female Pelvic Med Reconstr Surg 2014; 20: 83–86. 24566210 10.1097/SPV.0000000000000044

[37] Nelson EC , Gottlieb AH , Müller HG , Smith W , Ali MR , Vidovszky TJ . Robotic cholecystectomy and resident education: the UC Davis experience. Int J Med Robot 2014; 10: 218–222. 24307477 10.1002/rcs.1554

[38] Odermatt M , Ahmed J , Panteleimonitis S , Khan J , Parvaiz A . Prior experience in laparoscopic rectal surgery can minimise the learning curve for robotic rectal resections: a cumulative sum analysis. Surg Endosc 2017; 31: 4067–4076. 28271267 10.1007/s00464-017-5453-9

[39] Park JH , Lee J , Hakim NA , Kim HY , Kang SW , Jeong JJ *et al* Robotic thyroidectomy learning curve for beginning surgeons with little or no experience of endoscopic surgery. Head Neck 2015; 37: 1705–1711. 24986508 10.1002/hed.23824

[40] Park SS , Kim MC , Park MS , Hyung WJ . Rapid adaptation of robotic gastrectomy for gastric cancer by experienced laparoscopic surgeons. Surg Endosc 2012; 26: 60–67. 21789643 10.1007/s00464-011-1828-5

[41] Paulucci DJ , Krane LS , Hemal AK , Badani KK . Beyond the learning curve: robotic partial nephrectomy outcomes continue to improve with surgeon experience. J Urol 2016; 195: e476.

[42] Pietrabissa A , Sbrana F , Morelli L , Badessi F , Pugliese L , Vinci A *et al* Overcoming the challenges of single‐incision cholecystectomy with robotic single‐site technology. Arch Surg 2012; 147: 709–714. 22508669 10.1001/archsurg.2012.508

[43] Pulliam SJ , Weinstein MM , Wakamatsu MM . Minimally invasive apical sacropexy: a retrospective review of laparoscopic and robotic operating room experiences. Female Pelvic Med Reconstr Surg 2012; 18: 122–126. 22453324 10.1097/SPV.0b013e31824a3995

[44] Riikonen J , Kaipia A , Petas A , Horte A , Koskimäki J , Kähkönen E *et al* Initiation of robot‐assisted radical prostatectomies in Finland: impact on centralization and quality of care. Scand J Urol 2016; 50: 149–154. 26881411 10.3109/21681805.2016.1142471

[45] Sarkaria IS , Latif MJ , Bianco VJ , Bains MS , Rusch VW , Jones DR *et al* Early operative outcomes and learning curve of robotic assisted giant paraesophageal hernia repair. Int J Med Robot 2017; 13: e1730. 10.1002/rcs.1730 PMC5182165 26928955

[46] Schatlo B , Martinez R , Alaid A , von Eckardstein K , Akhavan‐Sigari R , Hahn A *et al* Unskilled unawareness and the learning curve in robotic spine surgery. Acta Neurochir (Wien) 2015; 157: 1819–1823. 26287268 10.1007/s00701-015-2535-0

[47] Shakir M , Boone BA , Polanco PM , Zenati MS , Hogg ME , Tsung A *et al* The learning curve for robotic distal pancreatectomy: an analysis of outcomes of the first 100 consecutive cases at a high‐volume pancreatic centre. HPB 2015; 17: 580–586. 25906690 10.1111/hpb.12412 PMC4474504

[48] Sivaraman A , Sanchez‐Salas R , Prapotnich D , Yu K , Olivier F , Secin FP *et al* Learning curve of minimally invasive radical prostatectomy: comprehensive evaluation and cumulative summation analysis of oncological outcomes. Urol Oncol 2017; 35: 149. 10.1016/j.urolonc.2016.10.015 28117215

[49] Sood A , Ghani KR , Ahlawat R , Modi P , Abaza R , Jeong W *et al* Application of the statistical process control method for prospective patient safety monitoring during the learning phase: robotic kidney transplantation with regional hypothermia (IDEAL phase 2a–b). Eur Urol 2014; 66: 371–378. 24631408 10.1016/j.eururo.2014.02.055

[50] Tasian GE , Wiebe DJ , Casale P . Learning curve of robotic assisted pyeloplasty for pediatric urology fellows. J Urol 2013; 190: 1622–1626. 23410982 10.1016/j.juro.2013.02.009 PMC3706499

[51] Tobis S , Venigalla S , Knopf JK , Scosyrev E , Erturk EN , Golijanin DJ *et al* Robot‐assisted partial nephrectomy: analysis of the first 100 cases from a single institution. J Robot Surg 2012; 6: 139–147. 27628277 10.1007/s11701-011-0284-5

[52] van der Poel HG , de Blok W , Tillier C , van Muilekom E . Robot‐assisted laparoscopic prostatectomy: nodal dissection results during the first 440 cases by two surgeons. J Endourol 2012; 26: 1618–1624. 22800183 10.1089/end.2012.0360

[53] Vidovszky TJ , Carr AD , Farinholt GN , Ho HS , Smith WH , Ali MR . Single‐site robotic cholecystectomy in a broadly inclusive patient population: a prospective study. Ann Surg 2014; 260: 134–141. 24169178 10.1097/SLA.0000000000000295

[54] White HN , Frederick J , Zimmerman T , Carroll WR , Magnuson JS . Learning curve for transoral robotic surgery: a 4‐year analysis. JAMA Otolaryngol Head Neck Surg 2013; 139: 564–567. 23680949 10.1001/jamaoto.2013.3007

[55] Woelk JL , Casiano ER , Weaver AL , Gostout BS , Trabuco EC , Gebhart JB . The learning curve of robotic hysterectomy. Obstet Gynecol 2013; 121: 87–95. 23262932 10.1097/aog.0b013e31827a029e

[56] Wolanski P , Chabert C , Jones L , Mullavey T , Walsh S , Gianduzzo T . Preliminary results of robot‐assisted laparoscopic radical prostatectomy (RALP) after fellowship training and experience in laparoscopic radical prostatectomy (LRP). BJU Int 2012; 110: 64–70. 23194128 10.1111/j.1464-410X.2012.11479.x

[57] Zhou J , Shi Y , Qian F , Tang B , Hao Y , Zhao Y *et al* Cumulative summation analysis of learning curve for robot‐assisted gastrectomy in gastric cancer. J Surg Oncol 2015; 111: 760–767. 25580709 10.1002/jso.23876

[58] Zureikat AH , Moser AJ , Boone BA , Bartlett DL , Zenati M , Zeh HJ III . 250 robotic pancreatic resections: safety and feasibility. Ann Surg 2013; 258: 554–562. 24002300 10.1097/SLA.0b013e3182a4e87c PMC4619895

[59] Bric J , Connolly M , Kastenmeier A , Goldblatt M , Gould JC . Proficiency training on a virtual reality robotic surgical skills curriculum. Surg Endosc 2014; 28: 3343–3348. 24946742 10.1007/s00464-014-3624-5

[60] Gomez PP , Willis RE , Van Sickle KR . Development of a virtual reality robotic surgical curriculum using the da Vinci Si surgical system. Surg Endosc 2015; 29: 2171–2179. 25361648 10.1007/s00464-014-3914-y

[61] Angelo RL , Ryu RK , Pedowitz RA , Beach W , Burns J , Dodds J *et al* A proficiency‐based progression training curriculum coupled with a model simulator results in the acquisition of a superior arthroscopic Bankart skill set. Arthroscopy 2015; 31: 1854–1871. 26341047 10.1016/j.arthro.2015.07.001

[62] Hopper AN , Jamison MH , Lewis WG . Learning curves in surgical practice. Postgrad Med J 2007; 83: 777–779. 18057179 10.1136/pgmj.2007.057190 PMC2750931

[63] Pernar LIM , Robertson FC , Tavakkoli A , Sheu EG , Brooks DC , Smink DS . An appraisal of the learning curve in robotic general surgery. Surg Endosc 2017; 31: 4583–4596. 28411345 10.1007/s00464-017-5520-2

[64] Yang K , Perez M , Hossu G , Hubert N , Perrenot C , Hubert J . ‘Alarm‐corrected’ ergonomic armrest use could improve learning curves of novices on robotic simulator. Surg Endosc 2017; 31: 100–106. 27189375 10.1007/s00464-016-4934-6

[65] Walliczek U , Förtsch A , Dworschak P , Teymoortash A , Mandapathil M , Werner J *et al* Effect of training frequency on the learning curve on the da Vinci Skills Simulator. Head Neck 2016; 38(Suppl 1): E1762–E1769. 26681572 10.1002/hed.24312

[66] Harrison P , Raison N , Abe T , Watkinson W , Dar F , Challacombe B *et al* The validation of a novel robot‐assisted radical prostatectomy virtual reality module. J Surg Educ 2018; 75: 758–766. 28974429 10.1016/j.jsurg.2017.09.005

[67] Jepsen P , Johnsen SP , Gillman MW , Sørensen HT . Interpretation of observational studies. Heart 2004; 90: 956–960. 15253985 10.1136/hrt.2003.017269 PMC1768356

[68] StatsDirect . *Prospective vs. Retrospective Studies* https://www.statsdirect.com/help/basics/prospective.htm [accessed 22 November 2018].

[69] Pusic MV , Boutis K , Pecaric MR , Savenkov O , Beckstead JW , Jaber MY . A primer on the statistical modelling of learning curves in health professions education. Adv Health Sci Educ Theory Pract 2017; 22: 741–759. 27699508 10.1007/s10459-016-9709-2

[70] Yelle LE . The learning curve: historical review and comprehensive survey. Decis Sci 1979; 10: 302–328.

[71] Feldman LS , Cao J , Andalib A , Fraser S , Fried GM . A method to characterize the learning curve for performance of a fundamental laparoscopic simulator task: defining ‘learning plateau’ and ‘learning rate’. Surgery 2009; 146: 381–386. 19628099 10.1016/j.surg.2009.02.021

[72] Adam MA , Thomas S , Youngwirth L , Hyslop T , Reed SD , Scheri RP *et al* Is there a minimum number of thyroidectomies a surgeon should perform to optimize patient outcomes? Ann Surg 2017; 265: 402–407. 28059969 10.1097/SLA.0000000000001688

[73] Satava RM , Gallagher AG , Pellegrini CA . Surgical competence and surgical proficiency: definitions, taxonomy, and metrics. J Am Coll Surg 2003; 196: 933–937. 12788431 10.1016/S1072-7515(03)00237-0

[74] Mendes da Costa T. Procedure‐based assessments: an appropriate assessment tool? Bull R Coll Surg England 2014; 96: 236–238.

[75] Khan N , Abboudi H , Khan MS , Dasgupta P , Ahmed K . Measuring the surgical ‘learning curve’: methods, variables and competency. BJU Int 2014; 113: 504–508. 23819461 10.1111/bju.12197

[76] Hung AJ , Chen J , Gill IS . Automated performance metrics and machine learning algorithms to measure surgeon performance and anticipate clinical outcomes in robotic surgery. JAMA Surg 2018; 153: 770–771. 29926095 10.1001/jamasurg.2018.1512 PMC9084629

