# Supplementary material for: Systematic review of learning curves in robot‐assisted surgery
Source: BJS Open. 2019 Nov 29;4(1):27–44. doi: 10.1002/bjs5.50235 (PMC6996634; doi:10.1002/bjs5.50235)
Supplement: Supplementary file 1 — Table S1. Search terms for MEDLINE, MEDLINE In‐Process, MEDLINE Epub Ahead of Print and Embase Table S2. Search terms for Cochrane Library databases (searched via Wiley Online platform) Table S3. Full eligibility criteria Table S4. List of studies excluded at full‐text review and reasons for exclusion Table S5. Quality assessment of non‐randomized articles using Downs and Black checklist [file BJS5-4-27-s001.docx]

**BJS5_50235**

**Systematic review of learning curves in robot-assisted surgery**

**N. A. Soomro, D. A. Hashimoto, A. J. Porteous, C. J. A. Ridley, W. J. Marsh, R. Ditto and S. Roy**

Table S1 Search terms for MEDLINE, MEDLINE In-Process, MEDLINE Epub Ahead of Print and Embase

| **Term groups** | **#** | **Terms** | **Hits** |
| --- | --- | --- | --- |
| **Robot-assisted surgery terms** | 1 | *robotics/ | 29 766 |
|  | 2 | exp Robotic Surgical Procedures/ | 5 145 |
|  | 3 | ((surgical or surgery) adj2 robot*).mp. | 32 480 |
|  | 4 | robot-assisted.mp. | 25 149 |
|  | 5 | robot*.mp. | 132 473 |
|  | 6 | or/1-5 | 132 474 |
| **Learning curve terms** | 7 | learning curve/ | 11 071 |
|  | 8 | learning curve.mp. | 59 597 |
|  | 9 | 7 or 8 | 59 602 |
|  | 10 | proficien*.mp. | 95 939 |
|  | 11 | train*.mp. | 2 065 520 |
|  | 12 | improvement.mp. | 2 186 203 |
|  | 13 | assessment.mp. | 4 674 706 |
|  | 14 | competen*.mp. | 667 461 |
|  | 15 | perform*.mp. | 10 050 278 |
|  | 16 | performance.mp. | 2 942 379 |
|  | 17 | skill.mp. | 275 632 |
|  | 18 | or/10-17 | 15 282 231 |
|  | 19 | 6 and 9 and 18 | 6 727 |
| **Exclusionary study design terms** | 20 | Conference abstract.pt. | 2 873 775 |
|  | 21 | limit 20 to yr="1860 - 2015" | 2 247 084 |
|  | 22 | exp Comment/ or comment.pt. | 702 797 |
|  | 23 | exp Editorial/ or editorial.pt. | 1 333 459 |
|  | 24 | exp Letter/ or letter.pt. | 2 450 206 |
|  | 25 | "Case reports".pt. | 1 863 736 |
|  | 26 | (case report$ or protocol$).ti. | 736 890 |
|  | 27 | or/21-26 | 8 402 135 |
| **Inclusionary study design terms** | 28 | Case-Control Studies/ or Control Groups/ or Matched-Pair Analysis/ or retrospective studies/ or ((case* adj5 control*) or (case adj3 comparison*) or control group*).ti,ab,kw. | 2 670 718 |
|  | 29 | cohort studies/ or longitudinal studies/ or follow-up studies/ or prospective studies/ or retrospective studies/ or cohort.ti,ab. or longitudinal.ti,ab. or prospective.ti,ab. or retrospective.ti,ab. | 5 448 313 |
|  | 30 | ("clinical trial" or "clinical trial, phase i" or "clinical trial, phase ii" or clinical trial, phase iii or clinical trial, phase iv or controlled clinical trial or "multicenter study" or "randomized controlled trial").pt. or double-blind method/ or clinical trials as topic/ or clinical trials, phase i as topic/ or clinical trials, phase ii as topic/ or clinical trials, phase iii as topic/ or clinical trials, phase iv as topic/ or controlled clinical trials as topic/ or randomized controlled trials as topic/ or early termination of clinical trials as topic/ or multicenter studies as topic/ or ((randomi?ed adj7 trial*) or (controlled adj3 trial*) or (clinical adj2 trial*) or ((single or doubl* or tripl* or treb*) and (blind* or mask*))).ti,ab,kw. or ("4 arm" or "four arm").ti,ab,kw. | 2 707 881 |
|  | 31 | Cross-Sectional Studies/ or Prevalence/ or (cross-sectional or prevalence or transversal).ti,ab,kw. | 2 236 930 |
|  | 32 | (((comprehensive* or integrative or systematic*) adj3 (bibliographic* or review* or literature)) or (meta-analy* or metaanaly* or "research synthesis" or ((information or data) adj3 synthesis) or (data adj2 extract*))).ti,ab. or (cinahl or (cochrane adj3 trial*) or embase or medline or psyclit or (psycinfo not "psycinfo database") or pubmed or scopus or "sociological abstracts" or "web of science").ab. or ("cochrane database of systematic reviews" or evidence report technology assessment or evidence report technology assessment summary).jn. or Evidence Report: Technology Assessment*.jn. or ((review adj5 (rationale or evidence)).ti,ab. and review.pt.) or meta-analysis as topic/ or Meta-Analysis.pt. | 890 517 |
|  | 33 | health economics/ or exp economic evaluation/ or cost-benefit analysis/ or cost effectiveness analysis/ or cost minimization analysis/ or cost utility analysis/ | 410 846 |
|  | 34 | (cost$ adj2 (effective$ or utilit$ or benefit$ or minimi$ or consequence$)).tw. | 596 234 |
|  | 35 | (quality adjusted life year$ or qaly$ or life year$ gained or life year$ equivalent$ or incremental cost effective* or icer).tw. | 66 896 |
|  | 36 | ((economic$ or pharmacoeconomic$) adj2 (evaluat* or model* or analys?s)).tw. | 88 803 |
|  | 37 | "Research Support, Non-U.S. Gov't".pt. | 6 609 368 |
| **Total** | 38 | or/28-37 | 16 575 137 |
|  | 39 | 19 and 38 | 3 188 |
|  | 40 | 39 not 27 | 2 715 |
|  | 41 | limit 40 to yr="2012-current" | 1 774 |

Table S2 Search terms for Cochrane Library databases (searched via Wiley Online platform)

| **Term groups** | **#** | **Terms** | **Hits** |
| --- | --- | --- | --- |
| **Robot-assisted surgery terms** | 1 | [mh robotics] | 765 |
|  | 2 | [mh "Robotic Surgical Procedures"] | 98 |
|  | 3 | ((surgical or surgery) near/2 (robot*)):ti,ab,kw | 741 |
|  | 4 | ("robot-assisted" or "robot assisted" or "robotic-assisted" or "robotic assisted") .mp. | 37 |
|  | 5 | robot*:ti,ab,kw | 2 435 |
|  | 6 | {or #1-#5} | 2 435 |
| **Learning curve terms** | 7 | [mh "Learning Curve"] | 139 |
|  | 8 | "learning curve":ti,ab,kw | 797 |
|  | 9 | {or #7-#8} | 797 |
|  | 10 | proficien*:ti,ab,kw | 950 |
|  | 11 | train*:ti,ab,kw | 57 109 |
|  | 12 | improvement:ti,ab,kw | 90 743 |
|  | 13 | assessment:ti,ab,kw | 136 352 |
|  | 14 | competen*:ti,ab,kw | 6 704 |
|  | 15 | perform*:ti,ab,kw | 194 567 |
|  | 16 | performance:ti,ab,kw | 61 920 |
|  | 17 | skill*:ti,ab,kw | 16 247 |
|  | 18 | {or #10-#17} | 381 953 |
| **Exclusionary study design terms** | 19 | [mh Comment] or comment:pt | 1 856 |
|  | 20 | [mh Editorial] or editorial:pt | 656 |
|  | 21 | [mh Letter] or letter:pt | 7 784 |
|  | 22 | "Case reports":pt | 1 535 |
|  | 23 | (case stud$ or case report$ or protocol$):ti | 12 182 |
| **Total** | 24 | {or #19-#23} | 22 469 |
|  | 25 | #6 and #9 | 84 |
|  | 26 | #25 and #18 | 76 |
|  | 27 | #26 not #24 | 75 |
|  | 28 | Limit #27 to 2012–2018 | 64 |

Table S3 Full eligibility criteria

| **Category** | **Inclusion criteria** | **Exclusion criteria** |
| --- | --- | --- |
| **Population** | - Surgeons* performing robot‑assisted surgery in any specialty - Surgeons* undergoing robot‑assisted surgical training on wet lab (animal/cadaveric), dry lab (models), or simulated (virtual reality) environments - No limits were placed on the patients undergoing robot‑assisted surgery | - Human, animal, cadaveric, biochemical, biomechanical or simulated procedures not using a surgical robot or simulator - Studies not in a real-life or simulated surgical setting - Studies specifically evaluating the learning curve of a single surgeon (alone or as part of a surgical team) |
| **Interventions** | - Any “master-and-slave” robot‑assisted surgical modality, in which metrics were reported separately for each procedure | - Studies not reporting a robot‑assisted surgical modality - Studies reporting metrics for multiple robot-assisted platforms without providing separate data for each platform - Studies reporting metrics for multiple robot-assisted procedures without providing separate data for each procedure |
| **Comparators** | - Any or no comparator | - No exclusion criteria |
| **Outcomes** | - Studies that present a graphical learning curve analysis and/or report learning data for at least 4 time points, performed using 1 of the following methods: - Any relevant variable^‡^ analysed over multiple procedures/tasks - ANOVA - CUSUM - Regression analysis - Moving average - LSE - MEM   AND:   - Reporting at least 1 of the following learning curve metrics: - Time to plateau/number of ‘phases’: any relevant variable assessed via a relevant technique^‡^ which allows the identification of time to plateau, competence, proficiency, procedures to overcome the learning curve or the number of phases - Difference in metrics over time: any relevant variable^‡^ assessed over time by splitting the procedures/tasks into groups and comparing the differences - Learning percentages: the change in any relevant variable^‡^ over time associated with each doubling of procedure/task number | - Studies that do not present a graphical learning curve analysis and/or report learning data for at least 4 time points, or do not present a relevant time to plateau/inflection analysis - Studies not including at least 1 relevant metric |
| **Study design** | - Randomised controlled trials, non-randomised interventional studies, observational studies - Perioperative clinical outcome evaluations - Non-clinical training or simulation evaluations - Registry analyses - Economic analyses | - Case reports or case series - Letters to the editor, commentaries, seminars - Narrative reviews - SLRs and meta-analyses (reference lists of relevant studies hand searched) |
| **Other considerations** | - Published in the English language - Articles published in or after 2012 - Conference abstracts published in or after 2016 - No limits on country of study - Studies with ≥20 total surgical procedures (not including training task studies) | - Not in the English language - Articles published before 2012 or conference abstracts published before 2016 - Conference abstract booklets not listed a priori - Studies with <20 total surgical procedures (not including training task studies) |

*Only studies that specifically evaluated the learning curve of a single surgeon were ineligible. Studies evaluating multiple surgeons (in any surgical approach), or studies that did not explicitly define how many surgeons were evaluated but which included multiple authors, were deemed eligible. ‡Relevant variables: any operative, procedural, recovery, safety or clinical outcome or process measure, including (but not limited to) operative time, blood loss, console time, warm ischemia time, length of stay, urinary continence.

**Abbreviations:** ANOVA: analysis of variance; CUSUM: cumulative sum; LSE: least square estimation; MEM: mixed effect model; SLR: systematic literature review.

Table S4 List of studies excluded at full-text review and reasons for exclusion

| **Author** | **Year** | **Reference** | **Reason for exclusion** |
| --- | --- | --- | --- |
| Abboudi et al. | 2014 | British Journal of Urology International; 114(4):617–629 | 5 |
| Abdi et al. | 2015 | Public Library of Science ONE; 10(7):e0134501 | 5 |
| Abe et al. | 2017 | Journal of Surgical Education; 75(2):458–464 | 6 |
| Acharya et al. | 2012 | Interactive Cardiovascular and Thoracic Surgery; 15(6):1040–1046 | 5 |
| Agrusa et al. | 2017 | Oncotarget; 8(60):102392–102400 | 5 |
| Ahmed et al. | 2012 | British Journal of Urology; 110(10):1544–1556 | 7 |
| Aldrighetti et al. | 2015 | Updates in Surgery; 67(2):129–140 | 5 |
| Alemozaffar et al. | 2012 | European Urology; 61(6):1222–1228 | 4 |
| Aliyev et al. | 2013 | Surgery; 153(5):705–710 | 5 |
| Al-Tartir et al. | 2015 | The Italian Journal of Urology and Nephrology; 67(1):55–63 | 3 |
| Altok et al. | 2018 | Urologic Oncology: Seminars and Original Investigations; 36(1):13 | 5 |
| Arora et al. | 2012 | Clinical Otolaryngology And Allied Sciences; 37:4-5 | 5 |
| Autorino et al. | 2014 | European Urology; 65(2):430–452 | 5 |
| Awad et al. | 2013 | Archives of Gynecology and Obstetrics; 287(6):1181–1186 | 4 |
| Aydin et al. | 2015 | British Journal of Urology International; 115(6):994–1003 | 2 |
| Ayloo et al. | 2014 | Surgical Endoscopy; 28(5):1629–1633 | 4 |
| Bai et al. | 2012 | Journal of Cardiovascular Electrophysiology; 23(8):820–826 | 2 |
| Bao et al. | 2014 | Journal of Gastrointestinal Surgery; 18(4):682–689 | 5 |
| Barrie et al. | 2014 | Annals of Surgical Oncology; 21(3):829–840 | 7 |
| Barrie et al. | 2015 | Surgical Laparoscopy Endoscopy and Percutaneous Techniques; 25(4):297–302 | 7 |
| Barth et al. | 2013 | Annals of Surgery; 257(3):527–533 | 2 |
| Bennich et al. | 2016 | Acta Obstetricia et Gynecologica Scandinavica; 95(8):894–900 | 2 |
| Bergamaschi et al. | 2017 | Surgical Endoscopy and Other Interventional Techniques; 31(2 Supplement 1):S58 | 5 |
| Beyer-Berjot et al. | 2014 | Surgery; 156(3):689–697 | 2 |
| Biler et al. | 2017 | Gazi Medical Journal; 28(2):93–97 | 5 |
| Bonaros et al. | 2013 | Annals of Thoracic Surgery; 95(3):803–812 | 5 |
| Brinkman et al. | 2015 | International Journal. Of Medical roboticsand Computer-Assisted Surgery; 11(3):308–318 | 5 |
| Broe et al. | 2017 | British Journal of Urology International; 120(Supplement 2):S32 | 5 |
| Brooks et al. | 2015 | Journal of the Association of perioperative Registered Nurses; 102(1):40–49 | 7 |
| Bruce et al. | 2016 | Journal of Urology; 195(4 Supplement 1):e217–e218 | 5 |
| Buckmire et al. | 2015 | The Laryngoscope; 125(6):1393–1400 | 2 |
| Burt et al. | 2015 | Journal of Thoracic and Cardiovascular Surgery; 150(5):1061–1067 | 2 |
| Cahill et al. | 2014 | Journal of Bone and Joint Surgery; 96(16):1333–1339 | 2 |
| Cao et al. | 2012 | Annals of Cardiothoracic Surgery; 1(1):43376 | 7 |
| Carter et al. | 2014 | British Journal Urology International; 113(5b):e112–e118 | 5 |
| Carter et al. | 2015 | Annals of Surgery; 261(5):870–875 | 5 |
| Catchpole et al. | 2016 | Surgical Endoscopy; 30(9):3749–3761 | 5 |
| Cela et al. | 2013 | Surgical Endoscopy; 27(7):2638–2643 | 4 |
| Chan et al. | 2016 | Laryngoscope; 126(3):566–569 | 5 |
| Cho et al. | 2013 | Journal of Laparoendoscopic & Advanced Surgical Techniques.; 23(12):992–998 | 5 |
| Coca-Soliz et al. | 2017 | Surgical Endoscopy and Other Interventional Techniques; 31(Supplement 1):S314 | 5 |
| Cohen et al. | 2015 | Ochsner Journal; 15(1):19–24 | 5 |
| Cundy et al. | 2015 | International Journal of Medical Robotics and Computer-Assisted Surgery; 11(2):141–149 | 4 |
| Davila et al. | 2017 | Journal of Robotic Surgery; 11(2):171–177 | 5 |
| De Castro Abreu et al. | 2014 | Indian Journal of Urology; 30(3):300–306 | 4 |
| de la Fuente et al. | 2013 | Surgical Endoscopy; 27(9):3339–3347 | 4 |
| DeLeon et al. | 2014 | American Journal of Gastroenterology; 109:S197 | 5 |
| Desai et al. | 2014 | Journal of Urology; 191(4 supplement 1):e85–e86 | 5 |
| Dev et al. | 2012 | British Journal of Urology International; 109(5):760–768 | 7 |
| Duchalais et al. | 2017 | Colorectal Disease; 19(Supplement 2):89 | 5 |
| Eddib et al. | 2013 | Journal of Robotic Surgery; 7(3):295–299 | 5 |
| El Hachem et al. | 2016 | International Journal of Medical Robotics and Computer-Assisted Surgery;12(3):509–516 | 4 |
| Ellison et al. | 2012 | Journal of Urology; 188(1):45–50 | 5 |
| Eriksen et al. | 2013 | Danish Medical Journal; 60(12) | 5 |
| Falkenback et al. | 2014 | ANZ Journal of Surgery; 84(10):712–721 | 5 |
| Fan et al. | 2012 | Chinese Medical Journal; 125(5):926–931 | 7 |
| Ferrara et al. | 2016 | Surgical Innovation; 23(4):374–380 | 4 |
| Ferriero et al. | 2016 | Journal of Endourology; 30(Supplement 2):A263 | 5 |
| Foley et al. | 2012 | Surgical Endoscopy 26(8):2259–2266 | 4 |
| Fourman et al. | 2012 | Surgery for Obesity and Related Diseases; 8(4):483–488 | 7 |
| Fransen et al. | 2015 | Surgical Endoscopy and Other Interventional Techniques.; 29:S231 | 5 |
| Gala et al. | 2014 | Journal of Minimally Invasive Gynecology; 21(3):353–361 | 7 |
| Galfano et al. | 2013 | European Urology; 64(6):974–980 | 4 |
| Galvani et al. | 2016 | Journal of Laparoendoscopic and Advanced Surgical Techniques; 26(4):290–295 | 4 |
| Geller et al. | 2013 | American Journal of Obstetrics and Gynecology; 209(1):20e1-20e5 | 5 |
| Grimminck et al. | 2016 | European Journal of Obstetrics Gynecology and Reproductive Biology; 206:27–31 | 4 |
| Hanafi et al. | 2016 | Gynecological Surgery; 13(1 Supplement 1):S87 | 5 |
| Hankins et al. | 2016 | International Urology and Nephrology; 48(11):1817–1821 | 5 |
| Hans et al. | 2012 | European Archives of Oto-Rhino-Laryngology; 269(8):1979–1984 | 4 |
| Hanzly et al. | 2015 | Journal of Endourology; 29(3):297–303 | 4 |
| Harrison et al. | 2015 | Journal of Surgical Oncology; 112(8):888–893 | 4 |
| Harrysson et al. | 2014 | Annals of Surgery; 260(1):37–45 | 7 |
| Harskamp et al. | 2015 | American Heart Journal; 169(4):557–563e6 | 5 |
| Herlemann et al. | 2017 | Journal of Urology; 197(4 Supplement 1):e284 | 5 |
| Herlemann et al. | 2018 | European Urology; 73(2):215–223 | 5 |
| Hinata et al. | 2014 | Urology; 83(5):1088–1092 | 5 |
| Holzhey et al. | 2013 | Circulation; 128(5):483–491 | 2 |
| Hoste et al. | 2015 | European Journal of Obstetrics Gynecology and Reproductive Biology; 194:241–244 | 4 |
| Housmanou et al. | 2017 | Interactive Cardiovascular and Thoracic Surgery; 25(Supplement 1): i68 | 5 |
| Jaiprakash et al. | 2017 | Journal of Orthopaedic Surgery; 25(1):1–6 | 2 |
| Jensen et al. | 2017 | International Journal of Surgery; 39:260–266 | 5 |
| Jimenez-Rodriguez et al. | 2013 | International Journal of Colorectal Disease; 28(6):815–821 | 4 |
| Kamara et al. | 2017 | Journal of Arthroplasty; 32(1):125–130 | 4 |
| Kandil et al. | 2012 | Archrives of Otolaryngology Head and Neck Surgery; 138(2):113–117 | 4 |
| Kang et al. | 2012 | Journal of Gastric Cancer.; 12:156–163. | 4 |
| Kang et al. | 2016 | Surgical Endoscopy and Other Interventional Techniques; 30(Supplement 1):S489 | 5 |
| Khan et al. | 2015 | Surgical Endoscopy and Other Interventional Techniques.; 29:S63 | 4 |
| Khan et al. | 2015 | British Journal of Urology International; 116(4):665–674 | 5 |
| Kim et al. | 2015 | Surgical Laparoscopy, Endoscopy & Percutaneous Techniques; 25(5):412–416 | 4 |
| Kiong et al. | 2015 | Otolaryngology Head and Neck Surgery; 152(5):820-826 | 5 |
| Klein et al. | 2016 | European Urology, Supplements; 15(3):e1032 | 5 |
| Ko et al. | 2017 | European Urology, Supplements; 16(3):e114–e115 | 5 |
| Kuo et al. | 2014 | International Journal of Colorectal Disease; 29:555–562 | 4 |
| Lai et al. | 2017 | Hepato-Biliary Surgery and Nutrition; 6(4):222–229 | 5 |
| Larsen et al. | 2012 | Acta Obstetricia Gynecologica Scandinavica; 91(9):1015–1028 | 5 |
| Laviana et al. | 2014 | World Journal of Urology; 32(3):591–596 | 3 |
| Leonardis et al. | 2013 | Otolaryngology Head and Neck Surgery; 149(upplement 2):P251 | 5 |
| Lin et al. | 2013 | Annals of Surgery; 257(2):205–213 | 5 |
| Liss et al. | 2013 | World Journal of Urology; 31(3):489–497 | 7 |
| Liu et al. | 2017 | Surgical Endoscopy and Other Interventional Techniques :43374 | 5 |
| Lorgat et al. | 2012 | Cardiovascular Journal of Africa; 23(5):274–280 | 2 |
| Lovegrove et al. | 2016 | Journal of Urology; 195(4 Supplement 1):e112 | 5 |
| Lovegrove et al. | 2017 | Journal of Surgical Education; 74(3):486–494 | 5 |
| Macdonald et al. | 2016 | Journal of laparoendoscopic & advanced surgical techniques; Part A 26(8):652–659 | 7 |
| Madec et al. | 2017 | Journal of Endourology; 31(7):655–660 | 2 |
| Madhuri et al. | 2012 | International Journal of Medical Robotics and Computer-Assisted Surgery;8(4):496–503 | 5 |
| Maenpaa et al. | 2015 | Acta Obstetricia et Gynecologica Scandinavica; 94(5):482–488 | 6 |
| Magistri et al. | 2017 | Journal of the American College of Surgeons; 225(4 Supplement 2):e127 | 5 |
| Marcovigi et al. | 2017 | Acta Biomedica; 88(Supplement 2):54–59 | 4 |
| Marino et al. | 2017 | Colorectal Disease; 19(Supplement 2):45–46 | 5 |
| Marino et al. | 2017 | Surgical Endoscopy and Other Interventional Techniques; 31(2 Supplement 1):S248 | 5 |
| Maruthappu et al. | 2015 | Annals of Surgery; 261(4):642–647 | 7 |
| Mathews et al. | 2017 | American Journal of Obstetrics and Gynecology; 217(5):596e1–596e7 | 5 |
| Maykel et al. | 2017 | Diseases of the Colon and Rectum; 60(12):1267–1272 | 5 |
| Mazzon et al. | 2017 | Current Urology Reports; 18(11):89 | 3 |
| Mietzsch et al. | 2016 | European Journal of Pediatric Surgery; 26(5):436–442 | 2 |
| Mirheydar et al. | 2013 | World Journal of Urology; 31(3):455–461 | 3 |
| Misa et al. | 2016 | Obstetrics and Gynecology; 127(Supplement 1):19S–20S | 5 |
| Miskovic et al. | 2012 | Diseases of the Colon and Rectum; 55(12):1300–1310 | 2 |
| Moghul et al. | 2013 | Surgical Innovation; 20(3):282–291 | 7 |
| Mohosho et al. | 2013 | World Journal of Laparoscopic Surgery ;6(3):163–166 | 3 |
| Mok et al. | 2012 | International Journal of Gynecological Cancer; 22(5):819–825 | 5 |
| Moon et al. | 2016 | Obesity Surgery; 26(10):2463–2468 | 4 |
| Moscarelli et al. | 2015 | International Journal of Medical Robotics and Computer-Assisted Surgery; 11(1):18–29 | 3 |
| Mourik et al. | 2012 | European Journal of Obstetrics Gynecology and Reproductive Biology; 165(1):122–127 | 4 |
| Mustafa-Michael et al. | 2016 | Female Pelvic Medicine and Reconstructive Surgery; 22(5 Supplement 1):S58 | 5 |
| Nagata et al. | 2016 | Surgery Today; 46(5):575–582 | 2 |
| Nakamura et al. | 2014 | Annals of Thoracic and Cardiovascular Surgery; 20(2):93–98 | 3 |
| Napoli et al. | 2015 | Updates in Surgery; 67(3):257–264 | 4 |
| Nassour et al. | 2016 | Journal of Clinical Oncology; 34(4 Supplement 1) | 5 |
| Nehme et al. | 2017 | Journal of Plastic, Reconstructive and Aesthetic Surgery Open; 13:1–10 | 5 |
| Neo et al. | 2016 | International Journal of Urology; 23(Supplement 1):39–40 | 5 |
| Nguyen et al. | 2017 | Journal of Thoracic Oncology; 12(11 Supplement 2):S2058–S2059 | 5 |
| Nic et al. | 2017 | British Journal of Urology International; 120(Supplement 2):50 | 5 |
| O'Connor et al. | 2017 | American Surgeon; 83(10):1085–1088 | 5 |
| Oh et al. | 2013 | The American Surgeon; 79(10):1075–1080 | 5 |
| O'Neill et al. | 2017 | Irish Journal of Medical Science; 186(8 Supplement 1):S331 | 5 |
| Ortenzi et al. | 2017 | Surgical Endoscopy and Other Interventional Techniques; 31(2 Supplement 1):S418 | 5 |
| Ozmen et al. | 2017 | Surgical Endoscopy and Other Interventional Techniques; 31(2 Supplement 1):S179 | 5 |
| Parekh et al. | 2013 | Journal of Urology; 189(2):474–479 | 5 |
| Parisi et al. | 2017 | Surgical Oncology; 26(1):28–36 | 4 |
| Park et al. | 2017 | European Journal of Cardio-Thoracic Surgery; 53(4):862–870 | 4 |
| Patel et al. | 2016 | Journal of Endourology.; 195(Supplement 2):A182 | 2 |
| Pernar et al. | 2016 | Surgical Endoscopy and Other Interventional Techniques; 30(Supplement 1):S269 | 3 |
| Pernar et al. | 2017 | Surgical Endoscopy; 31(11):4583–4596 | 7 |
| Perrenot et al. | 2013 | Diseases of the Colon and Rectum; 56(7):909–914 | 4 |
| Pilka et al. | 2017 | Ceska Gynekologie; 82(4):261–267 | 4 |
| Ponce et al. | 2016 | Journal of Minimally Invasive Gynecology; 23(4):622–627 | 5 |
| Porpiglia et al. | 2013 | British Journal of Urology international; 112(8):1125–1132 | 2 |
| Porpiglia et al. | 2016 | European Urology; 69(3):485–495 | 4 |
| Prado et al. | 2016 | Surgical Endoscopy and Other Interventional Techniques; 30(Supplement 1):S396 | 5 |
| Preece et al. | 2015 | Central European Journal of Urology; 68(2):207–211 | 5 |
| Pridgeon et al. | 2013 | British Journal of Urology International; 112(4):485–488 | 5 |
| Quarrier et al. | 2017 | Journal of Urology; 197(4 Supplement 1):e701–e702 | 5 |
| Quinn et al. | 2017 | Journal of the American College of Surgeons; 225(4 Supplement 2):e160–e161 | 5 |
| Raison et al. | 2016 | Journal of Urology; 195(4 Supplement 1):e115 | 5 |
| Raison et al. | 2017 | British Journal of Urology International; 119(5):804–811 | 5 |
| Ramzy et al. | 2014 | The Journal of Thoracic and Cardiovascular Surgery; 147(1):228–235 | 5 |
| Raque et al. | 2015 | Clinical Transplantation; 29(10):893–903 | 5 |
| Renaud et al. | 2013 | Obesity Surgery; 23(11):1753–1760 | 4 |
| Rhou et al. | 2015 | Australian and New Zealand Journal of Obstetrics and Gynaecology; 55(6):584–587 | 2 |
| Robertson et al. | 2013 | British Journal of Urology International; 112(6):798–812 | 7 |
| Samarasekera et al. | 2014 | Indian Journal of Urology; 30(3):293–299 | 5 |
| Scheib et al. | 2015 | American Journal of Obstetrics and Gynecology; 212(2):179e1–179e8 | 4 |
| Schlottmann et al. | 2017 | World Congress of Surgery; 27(7):661–665 | 5 |
| Schreuder et al. | 2012 | British Journal of Obstretrics and Gynaecology; 119(2):137–149 | 7 |
| Seco et al. | 2013 | Annals of Cardiothoracic Surgery; 2(4):408–418 | 7 |
| Selber et al. | 2012 | Plastic Reconstructive Surgery 130(4):550e–557e | 2 |
| Sendag et al. | 2014 | International Journal of Medical Robotics and Computer-Assisted Surgery; 10(3):275-279 | 4 |
| Serati et al. | 2014 | European Urology; 66(2):303–318 | 7 |
| Serrano et al. | 2017 | American Journal of Transplantation; 17(7):1868–1878 | 2 |
| Sharma et al. | 2016 | Journal of Minimally Invasive Gynecology; 23(1):89–93 | 4 |
| Shearer et al. | 2013 | Colorectal Disease; 15(10):1211–1226 | 2 |
| Sheth et al. | 2014 | Journal of Surgical Education; 71(1):125–132 | 5 |
| Shibasaki et al. | 2017 | Surgical Endoscopy and Other Interventional Techniques; 31(10):4283–4297 | 4 |
| Singla et al. | 2016 | ANZ Journal of Surgery; 86:115 | 5 |
| Sodhi et al. | 2017 | Journal of Knee Surgery; (no pagination) | 5 |
| Somashekhar et al. | 2017 | Journal of Minimal Access Surgery; 13(4):280–285 | 5 |
| Spinoglio et al. | 2012 | Surgical Endoscopy; 26(6):1648–1655 | 4 |
| Sridhar et al. | 2016 | European Urology Supplements. Conference: 13th Meeting of the EAU Robotic Urology Section 2016, Italy; 15(7):212 | 5 |
| Suero et al. | 2018 | International Journal of Medical Robotics and Computer-Assisted Surgery; 14(1):e1863 | 5 |
| Suh et al. | 2012 | Journal of Robotic Surgery; 6(4):301–309 | 5 |
| Taniguchi et al. | 2017 | Yonago Acta Medica; 60(3):162–166 | 5 |
| Tapper et al. | 2014 | European Journal of Obstetrics Gynecology and Reproductive Biology; 177:1–10 | 7 |
| Terris et al. | 2012 | Otolaryngology Head and Neck Surgery; 147(1):20–25 | 4 |
| Tobias-Machado et al. | 2016 | Journal of the Brazilian Society of Urology; 42(1):83–89 | 5 |
| Toker et al. | 2016 | Surgical Endoscopy; 30(2):676–683 | 4 |
| Tolley et al. | 2016 | Head & Neck; 38(Supplement 1):e300–e306 | 4 |
| Toro et al. | 2015 | Surgical Endoscopy; 29(1):1–8 | 3 |
| Tou et al. | 2017 | Colorectal Disease; 19(Supplement 2):S122 | 5 |
| Tourinho–Barbosa et al. | 2017 | Arab Journal of Urology; 16(3):285–292 | 7 |
| Tudor et al. | 2016 | Surgical Endoscopy and Other Interventional Techniques; 30(Supplement 1):S91 | 5 |
| Twijnstra et al. | 2012 | Obstetrics and Gynecology 119(4):700–708 | 2 |
| Valdis et al. | 2015 | Innovations: Technology and Techniques in Cardiothoracic and Vascular Surgery; 10:S9 | 5 |
| Van der Sluis et al. | 2016 | Diseases of the Esophagus; 29(Supplement 1):48A–49A | 5 |
| Van Hilst et al. | 2016 | United European Gastroenterology Journal; 4(5 Supplement 1):A377 | 5 |
| Veeratterapillay et al. | 2018 | British Journal of Urology International; 120(4):550–555 | 5 |
| Vergez et al. | 2012 | Otolaryngology Head and Neck Surgery; 147(3):475-481 | 5 |
| Walters et al. | 2014 | Journal of Robotic Surgery; 8(3):207-211 | 5 |
| Wang et al. | 2013 | Academic Journal of Second Military Medical University; 34(7):719-726 | 1 |
| Wilhelm et al. | 2016 | Translational Cancer Research; 5(Supplement 7) :S1521–S1530 | 5 |
| Wisa et al. | 2016 | BJOG: An International Journal of Obstetrics and Gynaecology; 123(Supplement 2):S222 | 3 |
| Wright et al. | 2016 | Journal of Gastrointestinal Surgery : Official Journal of the Society for Surgery of the Alimentary Tract; 20(9):1658–1665 | 7 |
| Xiao et al. | 2014 | Interactive Cardiovascular and Thoracic Surgery; 19(6):933–937 | 4 |
| Yan et al. | 2017 | Journal of Laparoendoscopic & Advanced Surgical Techniques; Part A 27(5):481–486 | 7 |
| Yang et al. | 2013 | National Medical Journal of China; 93(6):428–431 | 1 |
| Yang et al. | 2015 | Journal of Laparoendoscopic and Advanced Surgical Techniques; 25(12):1009–1018 | 5 |
| Yaribakht et al. | 2015 | Gynecologie Obstetrique et Fertilite; 43(5):348–355 | 1 |
| Yu et al. | 2017 | Surgical Endoscopy; and Other Interventional Techniques 32(3):1360-1367 | 5 |
| Zanagnolo et al. | 2017 | Journal of Minimally Invasive Gynecology; 24(3):379–396 | 3 |
| Zhang et al. | 2016 | Spine; 41:B23–B29 | 2 |

**Footnotes:** Reasons for exclusion included: 1 – Not published in English, or published before 2012 (if journal article) or 2016 (if conference abstract); 2 – Does not consider a relevant robot-assisted surgical procedure; 3 – Not of relevant design; 4 – Only includes a single surgeon/surgical team; 5 – Does not include a learning curve analysis; 6 – Does not report relevant learning curve metrics; 7 – Is an SLR or meta-analysis. SLR: systematic literature review.

Table S5 Quality assessment of non-randomized articles using Downs and Black checklist

| **Study** | **Question** | | | | | | | | | | | | | | | | | | | | | | | |
| --- | --- | --- | --- | --- | --- | --- | --- | --- | --- | --- | --- | --- | --- | --- | --- | --- | --- | --- | --- | --- | --- | --- | --- | --- |
|  | **1** | **2** | **3** | **4** | **5** | **6** | **7** | **8** | **9** | **10** | **11** | **12** | **13** | **14** | **15** | **16** | **17** | **18** | **20** | **21** | **22** | **25** | **26** | **27** |
| Albergotti et al. (2017)^10^ | ⬤ | ⬤ | ⬤ | ⬤ | ⬤ | ⬤ | ⬤ | ⬤ | - | ⬤ | ⬤ | ⬤ | ⬤ | ⬤ | ⬤ | ⬤ | - | ⬤ | ⬤ | ⬤ | ⬤ | ⬤ | - | ⬤ |
| Arora et al. (2017)^11^ | ⬤ | ⬤ | ⬤ | ⬤ | ⬤ | ⬤ | ⬤ | ⬤ | ⬤ | ⬤ | ⬤ | ⬤ | ⬤ | ⬤ | ⬤ | ⬤ | ⬤ | ⬤ | ⬤ | ⬤ | ⬤ | ⬤ | ⬤ | ⬤ |
| Benizri et al. (2014)^12^ | ⬤ | ⬤ | ⬤ | ⬤ | ⬤ | ⬤ | ⬤ | ⬤ | ⬤ | ⬤ | ⬤ | ⬤ | ⬤ | ⬤ | ⬤ | ⬤ | ⬤ | ⬤ | ⬤ | ⬤ | ⬤ | ⬤ | ⬤ | ⬤ |
| Bindal et al. (2015)^13^ | ⬤ | ⬤ | ⬤ | ⬤ | ⬤ | ⬤ | ⬤ | ⬤ | ⬤ | ⬤ | ⬤ | ⬤ | ⬤ | ⬤ | ⬤ | ⬤ | ⬤ | ⬤ | ⬤ | ⬤ | ⬤ | ⬤ | ⬤ | ⬤ |
| Binet et al. (2017)^14^ | ⬤ | ⬤ | ⬤ | ⬤ | ⬤ | ⬤ | ⬤ | ⬤ | ⬤ | ⬤ | ⬤ | ⬤ | ⬤ | ⬤ | ⬤ | ⬤ | ⬤ | ⬤ | ⬤ | ⬤ | ⬤ | ⬤ | ⬤ | ⬤ |
| Boone et al. (2015)^15^ | ⬤ | ⬤ | ⬤ | ⬤ | ⬤ | ⬤ | ⬤ | ⬤ | ⬤ | ⬤ | ⬤ | ⬤ | ⬤ | ⬤ | ⬤ | ⬤ | ⬤ | ⬤ | ⬤ | ⬤ | ⬤ | ⬤ | ⬤ | ⬤ |
| Chang et al. (2016)^16^ | ⬤ | ⬤ | ⬤ | ⬤ | ⬤ | ⬤ | ⬤ | ⬤ | ⬤ | ⬤ | ⬤ | ⬤ | ⬤ | ⬤ | ⬤ | ⬤ | ⬤ | ⬤ | ⬤ | ⬤ | ⬤ | ⬤ | ⬤ | ⬤ |
| Ciabatti et al. (2012)^17^ | ⬤ | ⬤ | ⬤ | ⬤ | ⬤ | ⬤ | ⬤ | ⬤ | - | ⬤ | ⬤ | ⬤ | ⬤ | ⬤ | ⬤ | ⬤ | ⬤ | ⬤ | ⬤ | ⬤ | ⬤ | ⬤ | ⬤ | ⬤ |
| D’Annibale et al. (2012)^18^ | ⬤ | ⬤ | ⬤ | ⬤ | ⬤ | ⬤ | ⬤ | ⬤ | - | ⬤ | ⬤ | ⬤ | ⬤ | ⬤ | ⬤ | ⬤ | - | ⬤ | ⬤ | ⬤ | ⬤ | ⬤ | - | ⬤ |
| Davis et al. (2014)^19^ | ⬤ | ⬤ | ⬤ | ⬤ | ⬤ | ⬤ | ⬤ | ⬤ | - | ⬤ | ⬤ | ⬤ | ⬤ | ⬤ | ⬤ | ⬤ | ⬤ | ⬤ | ⬤ | ⬤ | ⬤ | ⬤ | - | ⬤ |
| Dhir et al. (2016)^20^ | ⬤ | ⬤ | ⬤ | ⬤ | ⬤ | ⬤ | ⬤ | ⬤ | ⬤ | ⬤ | ⬤ | ⬤ | ⬤ | ⬤ | ⬤ | ⬤ | ⬤ | ⬤ | ⬤ | ⬤ | ⬤ | ⬤ | ⬤ | ⬤ |
| Esposito et al. (2017)^21^ | ⬤ | ⬤ | ⬤ | ⬤ | ⬤ | ⬤ | ⬤ | ⬤ | ⬤ | ⬤ | ⬤ | ⬤ | ⬤ | ⬤ | ⬤ | ⬤ | ⬤ | ⬤ | ⬤ | ⬤ | ⬤ | ⬤ | ⬤ | ⬤ |
| Fahim et al. (2017)^22^ | ⬤ | ⬤ | ⬤ | ⬤ | ⬤ | ⬤ | ⬤ | ⬤ | ⬤ | ⬤ | ⬤ | ⬤ | ⬤ | ⬤ | ⬤ | ⬤ | ⬤ | ⬤ | ⬤ | ⬤ | ⬤ | ⬤ | ⬤ | ⬤ |
| Fossati et al. (2017)^23^ | ⬤ | ⬤ | ⬤ | ⬤ | ⬤ | ⬤ | ⬤ | ⬤ | ⬤ | ⬤ | ⬤ | ⬤ | ⬤ | ⬤ | ⬤ | ⬤ | ⬤ | ⬤ | ⬤ | ⬤ | ⬤ | ⬤ | ⬤ | ⬤ |
| Geller et al. (2013)^24^ | ⬤ | ⬤ | ⬤ | ⬤ | ⬤ | ⬤ | ⬤ | ⬤ | - | ⬤ | ⬤ | ⬤ | ⬤ | ⬤ | ⬤ | ⬤ | - | ⬤ | ⬤ | ⬤ | ⬤ | ⬤ | - | ⬤ |
| Good et al. (2015)^25^ | ⬤ | ⬤ | ⬤ | ⬤ | ⬤ | ⬤ | ⬤ | ⬤ | ⬤ | ⬤ | ⬤ | ⬤ | ⬤ | ⬤ | ⬤ | ⬤ | ⬤ | ⬤ | ⬤ | ⬤ | ⬤ | ⬤ | ⬤ | ⬤ |
| Goodman et al. (2017)^26^ | ⬤ | ⬤ | ⬤ | ⬤ | ⬤ | ⬤ | ⬤ | ⬤ | ⬤ | ⬤ | ⬤ | ⬤ | ⬤ | ⬤ | ⬤ | ⬤ | ⬤ | ⬤ | ⬤ | ⬤ | ⬤ | ⬤ | ⬤ | ⬤ |
| Guend et al. (2017)^27^ | ⬤ | ⬤ | ⬤ | ⬤ | ⬤ | ⬤ | ⬤ | ⬤ | - | ⬤ | ⬤ | ⬤ | ⬤ | ⬤ | ⬤ | ⬤ | - | ⬤ | ⬤ | ⬤ | ⬤ | ⬤ | - | ⬤ |
| Kamel et al. (2017)^28^ | ⬤ | ⬤ | ⬤ | ⬤ | ⬤ | ⬤ | ⬤ | ⬤ | ⬤ | ⬤ | ⬤ | ⬤ | ⬤ | ⬤ | ⬤ | ⬤ | ⬤ | ⬤ | ⬤ | ⬤ | ⬤ | ⬤ | ⬤ | ⬤ |
| Kim et al. (2014)^29^ | ⬤ | ⬤ | ⬤ | ⬤ | ⬤ | ⬤ | ⬤ | ⬤ | ⬤ | ⬤ | ⬤ | ⬤ | ⬤ | ⬤ | ⬤ | ⬤ | ⬤ | ⬤ | ⬤ | ⬤ | ⬤ | ⬤ | ⬤ | ⬤ |
| Lebeau et al. (2012)^30^ | ⬤ | ⬤ | ⬤ | ⬤ | ⬤ | ⬤ | ⬤ | ⬤ | - | ⬤ | ⬤ | ⬤ | ⬤ | ⬤ | ⬤ | ⬤ | ⬤ | ⬤ | ⬤ | ⬤ | ⬤ | ⬤ | ⬤ | ⬤ |
| Linder et al. (2016)^31^ | ⬤ | ⬤ | ⬤ | ⬤ | ⬤ | ⬤ | ⬤ | ⬤ | ⬤ | ⬤ | ⬤ | ⬤ | ⬤ | ⬤ | ⬤ | ⬤ | ⬤ | ⬤ | ⬤ | ⬤ | ⬤ | ⬤ | ⬤ | ⬤ |
| Lopez et al. (2016)^32^ | ⬤ | ⬤ | ⬤ | ⬤ | ⬤ | ⬤ | ⬤ | ⬤ | ⬤ | ⬤ | ⬤ | ⬤ | ⬤ | ⬤ | ⬤ | ⬤ | ⬤ | ⬤ | ⬤ | ⬤ | ⬤ | ⬤ | ⬤ | ⬤ |
| Lovegrove et al. (2016)^33^ | ⬤ | ⬤ | ⬤ | ⬤ | ⬤ | ⬤ | ⬤ | ⬤ | ⬤ | ⬤ | ⬤ | ⬤ | ⬤ | - | ⬤ | ⬤ | ⬤ | ⬤ | ⬤ | ⬤ | ⬤ | ⬤ | ⬤ | ⬤ |
| Luciano et al. (2016)^34^ | ⬤ | ⬤ | ⬤ | ⬤ | ⬤ | ⬤ | ⬤ | ⬤ | - | ⬤ | ⬤ | ⬤ | ⬤ | ⬤ | ⬤ | ⬤ | ⬤ | ⬤ | ⬤ | ⬤ | ⬤ | ⬤ | - | ⬤ |
| Meyer et al. (2012)^35^ | ⬤ | ⬤ | ⬤ | ⬤ | ⬤ | ⬤ | ⬤ | ⬤ | - | ⬤ | ⬤ | ⬤ | ⬤ | ⬤ | ⬤ | ⬤ | - | ⬤ | ⬤ | ⬤ | ⬤ | ⬤ | - | ⬤ |
| Myers et al. (2014)^36^ | ⬤ | ⬤ | ⬤ | ⬤ | ⬤ | ⬤ | ⬤ | ⬤ | - | ⬤ | ⬤ | ⬤ | ⬤ | ⬤ | ⬤ | ⬤ | - | ⬤ | ⬤ | ⬤ | ⬤ | ⬤ | - | ⬤ |
| Nelson et al. (2014)^37^ | ⬤ | ⬤ | ⬤ | ⬤ | ⬤ | ⬤ | ⬤ | ⬤ | - | ⬤ | ⬤ | ⬤ | ⬤ | ⬤ | ⬤ | ⬤ | - | ⬤ | ⬤ | ⬤ | ⬤ | ⬤ | - | ⬤ |
| Odermatt et al. (2017)^38^ | ⬤ | ⬤ | ⬤ | ⬤ | ⬤ | ⬤ | ⬤ | ⬤ | ⬤ | ⬤ | ⬤ | ⬤ | ⬤ | ⬤ | ⬤ | ⬤ | ⬤ | ⬤ | ⬤ | ⬤ | ⬤ | ⬤ | ⬤ | ⬤ |
| Park et al. (2012)^40^ | ⬤ | ⬤ | ⬤ | ⬤ | ⬤ | ⬤ | ⬤ | ⬤ | ⬤ | ⬤ | ⬤ | ⬤ | ⬤ | ⬤ | ⬤ | ⬤ | ⬤ | ⬤ | ⬤ | ⬤ | ⬤ | ⬤ | ⬤ | ⬤ |
| Park et al. (2015)^39^ | ⬤ | ⬤ | ⬤ | ⬤ | ⬤ | ⬤ | ⬤ | ⬤ | ⬤ | ⬤ | ⬤ | ⬤ | ⬤ | ⬤ | ⬤ | ⬤ | ⬤ | ⬤ | ⬤ | ⬤ | ⬤ | ⬤ | ⬤ | ⬤ |
| Paulucci et al. (2016)^41^ | ⬤ | ⬤ | ⬤ | ⬤ | ⬤ | ⬤ | ⬤ | - | ⬤ | ⬤ | ⬤ | ⬤ | ⬤ | ⬤ | ⬤ | - | ⬤ | ⬤ | ⬤ | ⬤ | ⬤ | - | ⬤ | ⬤ |
| Pietrabissa et al. (2012)^42^ | ⬤ | ⬤ | ⬤ | ⬤ | ⬤ | ⬤ | ⬤ | ⬤ | ⬤ | ⬤ | ⬤ | ⬤ | ⬤ | ⬤ | ⬤ | ⬤ | ⬤ | ⬤ | ⬤ | ⬤ | ⬤ | - | ⬤ | ⬤ |
| Pulliam et al. (2012)^43^ | ⬤ | ⬤ | ⬤ | ⬤ | ⬤ | ⬤ | ⬤ | - | ⬤ | ⬤ | ⬤ | ⬤ | ⬤ | ⬤ | ⬤ | ⬤ | ⬤ | ⬤ | ⬤ | ⬤ | ⬤ | ⬤ | ⬤ | ⬤ |
| Riikonen et al. (2016)^44^ | ⬤ | ⬤ | ⬤ | ⬤ | ⬤ | ⬤ | ⬤ | ⬤ | ⬤ | ⬤ | ⬤ | ⬤ | ⬤ | ⬤ | ⬤ | ⬤ | ⬤ | ⬤ | ⬤ | ⬤ | ⬤ | ⬤ | ⬤ | ⬤ |
| Sarkaria et al. (2017)^45^ | ⬤ | ⬤ | ⬤ | ⬤ | ⬤ | ⬤ | ⬤ | ⬤ | ⬤ | ⬤ | ⬤ | ⬤ | ⬤ | ⬤ | ⬤ | ⬤ | ⬤ | ⬤ | ⬤ | ⬤ | ⬤ | ⬤ | ⬤ | ⬤ |
| Schatlo et al. (2015)^46^ | ⬤ | ⬤ | ⬤ | ⬤ | ⬤ | ⬤ | ⬤ | - | ⬤ | ⬤ | ⬤ | ⬤ | ⬤ | ⬤ | ⬤ | - | ⬤ | ⬤ | ⬤ | ⬤ | ⬤ | - | ⬤ | ⬤ |
| Shakir et al. (2015)^47^ | ⬤ | ⬤ | ⬤ | ⬤ | ⬤ | ⬤ | ⬤ | ⬤ | ⬤ | ⬤ | ⬤ | ⬤ | ⬤ | ⬤ | ⬤ | ⬤ | ⬤ | ⬤ | ⬤ | ⬤ | ⬤ | ⬤ | ⬤ | ⬤ |
| Sivaraman et al. (2017)^48^ | ⬤ | ⬤ | ⬤ | ⬤ | ⬤ | ⬤ | ⬤ | ⬤ | ⬤ | ⬤ | ⬤ | ⬤ | ⬤ | ⬤ | ⬤ | ⬤ | ⬤ | ⬤ | ⬤ | ⬤ | ⬤ | ⬤ | ⬤ | ⬤ |
| Sood et al. (2014)^49^ | ⬤ | ⬤ | ⬤ | ⬤ | ⬤ | ⬤ | ⬤ | ⬤ | ⬤ | ⬤ | ⬤ | ⬤ | ⬤ | ⬤ | ⬤ | ⬤ | ⬤ | ⬤ | ⬤ | ⬤ | ⬤ | ⬤ | ⬤ | ⬤ |
| Tasian et al. (2013)^50^ | ⬤ | ⬤ | ⬤ | ⬤ | ⬤ | ⬤ | ⬤ | ⬤ | ⬤ | ⬤ | ⬤ | ⬤ | ⬤ | ⬤ | ⬤ | ⬤ | ⬤ | ⬤ | ⬤ | ⬤ | ⬤ | ⬤ | ⬤ | ⬤ |
| Tobis et al. (2012)^51^ | ⬤ | ⬤ | ⬤ | ⬤ | ⬤ | ⬤ | ⬤ | ⬤ | ⬤ | ⬤ | ⬤ | ⬤ | ⬤ | ⬤ | ⬤ | ⬤ | ⬤ | ⬤ | ⬤ | ⬤ | ⬤ | ⬤ | ⬤ | ⬤ |
| van der Poel et al. (2012)^52^ | ⬤ | ⬤ | ⬤ | ⬤ | ⬤ | ⬤ | ⬤ | ⬤ | ⬤ | ⬤ | ⬤ | ⬤ | ⬤ | ⬤ | ⬤ | ⬤ | ⬤ | ⬤ | ⬤ | ⬤ | ⬤ | ⬤ | ⬤ | ⬤ |
| Vidovszky et al. (2014)^53^ | ⬤ | ⬤ | ⬤ | ⬤ | ⬤ | ⬤ | ⬤ | ⬤ | ⬤ | ⬤ | ⬤ | ⬤ | ⬤ | ⬤ | ⬤ | ⬤ | ⬤ | ⬤ | ⬤ | ⬤ | ⬤ | ⬤ | ⬤ | ⬤ |
| White et al. (2013)^54^ | ⬤ | ⬤ | ⬤ | ⬤ | ⬤ | ⬤ | ⬤ | ⬤ | ⬤ | ⬤ | ⬤ | ⬤ | ⬤ | ⬤ | ⬤ | ⬤ | ⬤ | ⬤ | ⬤ | ⬤ | ⬤ | ⬤ | ⬤ | ⬤ |
| Woelk et al. (2013)^55^ | ⬤ | ⬤ | ⬤ | ⬤ | ⬤ | ⬤ | ⬤ | ⬤ | ⬤ | ⬤ | ⬤ | ⬤ | ⬤ | ⬤ | ⬤ | ⬤ | ⬤ | ⬤ | ⬤ | ⬤ | ⬤ | ⬤ | ⬤ | ⬤ |
| Wolanski et al. (2012)^56^ | ⬤ | ⬤ | ⬤ | ⬤ | ⬤ | ⬤ | ⬤ | ⬤ | ⬤ | ⬤ | ⬤ | ⬤ | ⬤ | ⬤ | ⬤ | ⬤ | ⬤ | ⬤ | ⬤ | ⬤ | ⬤ | ⬤ | ⬤ | ⬤ |
| Zhou et al. (2015)^57^ | ⬤ | ⬤ | ⬤ | ⬤ | ⬤ | ⬤ | ⬤ | ⬤ | ⬤ | ⬤ | ⬤ | ⬤ | ⬤ | ⬤ | ⬤ | ⬤ | ⬤ | ⬤ | ⬤ | ⬤ | ⬤ | ⬤ | ⬤ | ⬤ |
| Zureikat et al. (2013)^58^ | ⬤ | ⬤ | ⬤ | ⬤ | ⬤ | ⬤ | ⬤ | ⬤ | ⬤ | ⬤ | ⬤ | ⬤ | ⬤ | ⬤ | ⬤ | ⬤ | ⬤ | ⬤ | ⬤ | ⬤ | ⬤ | ⬤ | ⬤ | ⬤ |

**Footnotes:** ⬤ Lower risk of bias; ⬤ Higher risk of bias; ⬤ Risk of bias could not be determined from the publication (unclear); - Not applicable.
